# Review on 3D Printing Filaments Used in Fused Deposition Modeling Method for Dermatological Preparations

**DOI:** 10.3390/molecules30112411

**Published:** 2025-05-30

**Authors:** Yong Li Chan, Riyanto Teguh Widodo, Long Chiau Ming, Abdullah Khan, Syed Atif Abbas, Ng Yen Ping, Zarif Mohamed Sofian, Mahibub Mahamadsa Kanakal

**Affiliations:** 1 Department of Pharmaceutical Technology, Faculty of Pharmacy, Universiti Malaya, Kuala Lumpur 50603, Malaysia; ashleychan0819@gmail.com (Y.L.C.); riyanto@um.edu.my (R.T.W.); ms_zarif@um.edu.my (Z.M.S.); 2 Datta Meghe College of Pharmacy, Datta Meghe Institute of Higher Education (Deemed to be University), Sawangi (M), Wardha 442004, India; 3 Faculty of Medical and Life Sciences, Sunway University, Sunway City 47500, Malaysia; 4 Faculty of Pharmacy, Quest International University, Ipoh 30250, Malaysia; abdullah.khan@qiu.edu.my (A.K.); syed.atif@qiu.edu.my (S.A.A.); yenpingng@hotmail.com (N.Y.P.)

**Keywords:** polymers, filaments, 3D printing, fused deposition modeling (FDM), dermatology preparations, topical

## Abstract

Three-dimensional printing, particularly Fused Deposition Modeling (FDM), has revolutionized dermatological drug delivery by offering the ability to create personalized and precise drug formulations. This technology enables the design of customized drug delivery systems using a variety of polymers, such as Polylactic Acid (PLA), Polyvinyl Alcohol (PVA), Polyethylene Glycol (PEG), and Polycaprolactone (PCL), each with unique properties that enhance drug release, patient compliance, and treatment efficacy. This review analyzes these polymers in terms of their advantages, limitations, and suitability for dermatological applications. The ability to tailor these materials offers significant potential in overcoming treatment regimens. Additionally, the customization of three-dimensional-printed drug delivery systems provides a platform for creating patient-specific solutions that are more effective and adaptable to individual needs. Despite challenges such as moisture sensitivity and mechanical brittleness, the potential of FDM technology to improve dermatological treatments remains promising. The future of three-dimensional printing in dermatology lies in the integration of optimized materials and advanced printing techniques, which could further enhance patient-specific care and broaden the clinical applicability of these technologies in the pharmaceutical and biomedical sectors. By addressing these limitations and expanding material choices, FDM-based drug delivery systems have the potential to revolutionize the management of dermatological conditions, offering improved therapeutic outcomes and quality of life for patients.

## 1. Background

The integration of 3D printing technology, particularly Fused Deposition Modeling (FDM), has revolutionized the field of dermatological drug delivery. This advancement offers unparalleled potential for the customization of drug delivery systems, addressing specific patient needs while optimizing therapeutic outcomes. Dermatological conditions, affecting a significant portion of the global population, present diverse challenges ranging from treatment adherence to drug delivery efficiency. Traditional topical therapies are limited by the skin’s barrier properties, requiring novel solutions for improved drug penetration and sustained release.

Existing literature underscores the potential of 3D printing in healthcare, with FDM emerging as a versatile technique for creating drug-loaded devices [1]. Polymers such as PLA, PVA, PCL, and chitosan have been explored for their unique properties in fabricating dermatological preparations. However, there remains a gap in systematically evaluating the suitability of various FDM-compatible polymers for dermatological applications, particularly in overcoming limitations such as moisture sensitivity, mechanical brittleness, and drug delivery efficiency.

This review aims to bridge this gap by providing a comprehensive analysis of filaments used in FDM for dermatological preparations by examining the properties, drug-loading capacities, and practical applications of these materials. The insights gained here are poised to support the broader adoption of FDM technology in dermatology, fostering innovation and improving patient outcomes.

## 2. Introduction

Three-dimensional printing technology has revolutionized the healthcare fields including pharmaceutical science, offering innovative solutions for personalized medicine [2]. In dermatology, 3D printing enables the creation of customized drug delivery systems that can be tailored to disease conditions and individual patient requirements. A study demonstrated that 3D-printed microneedle arrays enhance transdermal drug delivery by improving skin penetration and controlled drug release, while personalized wound dressings fabricated via 3D printing provide optimized hydration and drug release profiles for enhanced healing [1]. This review focuses on the filaments used in 3D printing for topical drug delivery systems by the Fused Deposition Modeling Method, examining their properties, drug-loading capacities, and their applications.

### 2.1. The Impact of Dermatological Diseases and Disorders

Dermatological disorders represent a substantial portion of the global burden of disease, affecting individuals across various age groups and demographics. These conditions range from common ailments like acne and eczema to more severe disorders such as psoriasis. The prevalence of skin diseases is widespread and continues to increase worldwide, creating profound implications for physical health, quality of life, and healthcare systems [3].

A comprehensive report from the Global Burden of Disease Study (GBD) indicated that approximately 1.9 billion individuals, or nearly 25% of the global population, are affected by at least one skin disease at any given time [4]. Acne vulgaris, which predominantly affects adolescents, has been identified as one of the most prevalent dermatological conditions, with a global incidence rate of approximately 9.4% [5]. Eczema (atopic dermatitis) affects 15–20% of children and 1–3% of adults worldwide, making it another major concern in dermatology [6]. Psoriasis, a chronic autoimmune disorder, affects around 2–4% of the global population, equating to more than 125 million individuals [7]. Melanoma of the skin accounted for approximately 287,723 new cases and 60,712 deaths worldwide in 2018, making it the 19th most commonly diagnosed cancer globally [8]. In developing countries, infectious skin diseases such as scabies and impetigo remain highly prevalent, often driven by poor hygiene and overcrowded living conditions. For instance, the World Health Organization (WHO) estimates that over 200 million people globally suffer from scabies annually, with the majority residing in tropical and subtropical regions.

The widespread prevalence of dermatological disorders has significant ramifications across multiple facets of society. The impact on physical health is often compounded by severe psychological distress, as many skin conditions, such as psoriasis and eczema, are highly visible, leading to stigmatization, low self-esteem, and mental health issues, including anxiety and depression. Nearly 60% of individuals with psoriasis report that the disease poses a substantial problem in their daily lives while approximately one-third of people living with psoriasis experience depression. These statistics underscore the profound impact of psoriasis on both physical and psychological well-being [9].

Economically, skin diseases impose a considerable financial burden on healthcare systems and patients. In the United States alone, the direct medical costs associated with treating skin diseases are estimated to be over USD 75 billion annually [10]. Indirect costs, including lost productivity and absenteeism, further exacerbate the economic impact. For instance, individuals suffering from severe eczema may need to take time off work to manage flare-ups or attend medical appointments, leading to significant productivity losses [11]. In developing countries, the burden is amplified by limited access to dermatological care, resulting in inadequate treatment and prolonged suffering, particularly for preventable conditions like scabies and fungal infections [12].

### 2.2. Background on Topical Drug Delivery Systems

Topical drug therapy remains one of the most common and effective approaches to treating dermatological disorders. It has several advantages, including targeted delivery, reduced systemic side effects, and ease of application, making them an essential part of dermatological management. Various classes of topical agents are used depending on the type and severity of the condition, ranging from corticosteroids and antibiotics to immunomodulators and retinoids [13,14,15].

Topical drug delivery systems have traditionally relied on formulations such as creams and gels, designed for localized treatment of conditions like skin infections, inflammation, and pain [13,16]. These formulations are widely used due to their non-invasive nature and convenience. The stratum corneum, the outermost layer of the skin, serves as a substantial barrier, limiting the effectiveness of many topical therapies. Studies have shown that drug delivery through the skin is influenced by multiple factors, including formulation type, the presence of permeation enhancers, and the physicochemical properties of the drug [17]. As a result, achieving therapeutic drug concentrations in target tissues often requires high drug loading, which may increase the risk of side effects [18]. Furthermore, the variability in skin permeability between patients and different application sites makes it difficult to achieve consistent therapeutic outcomes [19].

In recent years, advancements in topical drug formulations have led to the development of new delivery systems, such as nanocarriers and 3D-printed transdermal patches, which enhance drug penetration and provide more consistent release profiles. Such innovations are particularly promising for the treatment of chronic conditions like psoriasis, where effective drug delivery to deeper skin layers is often challenging.

### 2.3. Emergence of Three-Dimensional (3D) Printing in Pharmaceuticals

Three-dimensional printing dates to 1984, when Charles W. Hull filed the first patent for stereolithography, a process to produce three-dimensional objects by layering materials based on Computer-Aided Design (CAD) models [20]. Initially, this technology served industrial purposes, facilitating rapid prototyping due to its speed, precision, and capability to produce highly customized models efficiently. Over the subsequent decades, 3D printing expanded significantly, transitioning from exclusively industrial use to broader applications across various sectors, driven by declining costs and enhanced accessibility to consumers and hobbyists [20].

In healthcare, 3D printing had significant advancements beginning in the early 2000s. Early trials at Boston Children’s Hospital, Harvard Medical School, involved manually constructing urinary bladders using scaffolds and patient cells, highlighting the potential for tissue engineering. This manual process prompted further automation, leading Anthony Atala to establish the Wake Forest Institute for Regenerative Medicine in 2004, where foundational research on bioprinting commenced using modified inkjet printers to fabricate customized organ scaffolds [20]. The medical field rapidly adopted 3D printing, utilizing it for surgical models, custom implants, and prosthetics, drastically enhancing precision in clinical outcomes. Landmark successes include titanium facial implants, customized hip replacements, jawbones, and even skull implants [20].

Contemporary developments have progressed towards bioprinting actual tissues and organs, demonstrating groundbreaking innovations like Organovo’s fully cellular 3D bioprinted liver tissues in 2013, paving the way for future transplantable organs [20]. Although fully functioning bioprinted organs remain an aspiration, the ongoing research and clinical trials underscore the transformative and expanding role of 3D printing in modern healthcare [20].

Today, 3D printing has transformed healthcare, including pharmaceuticals. Initially developed for rapid prototyping, it now enables precise fabrication of medical devices, implants, and personalized drug delivery systems [21]. The key benefit of 3D printing in pharmaceuticals is its ability to produce customized drug products tailored to individual needs, ensuring precise drug release [22]. It also allows for complex drug formulations that control release rates, improve bioavailability, and enhance patient compliance [23].

In dermatology, 3D printing revolutionizes drug delivery by enabling personalized and precise treatments. In contrast to conventional systems limited by mass production, 3D printing allows for custom formulations, addressing varied dosing needs and improving adherence [23]. It also enables intricate designs, beneficial for microneedle patches, transdermal systems, and wound dressings, improving targeted delivery [24]. Fused Deposition Modeling (FDM) provides a simple method to produce patient-specific drug devices. Another study showed that FDM-created custom drug patches to enhance treatment by ensuring precise application to the affected areas [24].

Conventional topical therapies struggle with limited skin penetration due to the stratum corneum, the skin’s natural barrier. Many APIs fail to reach therapeutic concentrations in deeper layers [16]. Three-dimensional-printed microneedle arrays, made using SLA or other methods, bypass this barrier, allowing for better drug penetration and bioavailability [25]. Moreover, 3D printing ensures controlled drug release. By designing complex geometries and multilayer structures, multiple APIs with different release profiles can be incorporated into a single device [26]. This enables immediate and sustained release, eliminating the need for frequent application.

Patient adherence is a major challenge in dermatology, especially with frequent application of topical treatments. Three-dimensional-printed devices provide a convenient, user-friendly alternative, requiring less frequent use [23]. Printed hydrogels and transdermal patches release drugs over extended periods, reducing reapplication and improving compliance [27]. Additionally, customized device designs ensure better fit and comfort, further enhancing patient experience.

Three-dimensional printing also allows for precise dosing and targeted delivery, minimizing drug waste and reducing side effects from systemic absorption [28]. On-demand manufacturing eliminates large-scale production and inventory costs, potentially lowering healthcare expenses [29].

Furthermore, 3D printing optimizes drug release by controlling the microstructure of materials, ensuring immediate, sustained, or controlled release as needed [30]. This ability to tailor release profiles improves treatment outcomes, particularly for localized and sustained therapies.

Beyond dermatological applications, 3D printing has revolutionized various biomedical fields, including prosthetics, organ modeling, and pharmaceutical manufacturing by enabling precise customization, improved patient-specific solutions, and efficient production workflows [31]. In prosthetics, 3D printing allows for the fabrication of highly customized, lightweight, and cost-effective prosthetic limbs tailored to individual patient needs, significantly improving comfort and functionality compared to traditional prosthetic fabrication methods. For organ modeling, 3D-printed anatomical structures are used for pre-surgical planning, medical training, and patient-specific implants, facilitating better surgical outcomes and reducing intraoperative risks [28].

In pharmaceutical manufacturing, 3D printing has paved the way for the development of personalized drug formulations, enabling the fabrication of dosage forms with controlled drug release profiles, complex geometries, and the ability to combine multiple active ingredients into a single unit [32]. Technologies such as Fused Deposition Modeling (FDM), Stereolithography (SLA), and Selective Laser Sintering (SLS) have been extensively explored for creating oral tablets, transdermal patches, and implantable drug delivery systems with enhanced therapeutic efficiency [33]. The introduction of patient-specific 3D-printed medications, such as the FDA-approved Spritam^®^ (levetiracetam), exemplifies how this technology can enhance drug bioavailability and patient adherence [34].

The ability to fabricate personalized implants and customized drug release systems underscores the versatility of 3D printing in modern healthcare, reducing manufacturing costs, minimizing waste, and enhancing treatment efficacy through precision medicine [28]. As research advances, the integration of bioinks, bioprinting techniques, and smart materials into 3D printing is expected to further expand its applications, particularly in tissue engineering and regenerative medicine. The potential of 3D printing also extends to pediatric and geriatric medicine, where customized doses, flavors, and dosage forms can improve patient acceptability and compliance [26]. The table below (Table 1) illustrates the milestones of 3D printing up until year 2024.

### 2.4. Overview of Three-Dimensional (3D) Printing Methods for Dermatological Applications

- Fused Deposition Modeling (FDM):
  ▪ Extrusion of thermoplastic polymers through a heated nozzle to build layers.
  ▪ Commonly used for creating drug-loaded patches and transdermal systems.
  ▪ Advantages: Cost-effective, versatile, and suitable for heat-sensitive APIs with low processing temperatures.
- Stereolithography (SLA):
  ▪ Utilizes UV lasers to cure photosensitive resins layer by layer.
  ▪ Ideal for high-resolution applications like microneedle arrays.
  ▪ Advantages: Produces intricate designs with sharp details and smooth surfaces.
- Selective Laser Sintering (SLS):
  ▪ Fuses powdered material using a laser to form layers.
  ▪ Useful for creating porous structures, enhancing drug loading and release.
  ▪ Advantages: No need for support structures, good for transdermal patches and wound dressings.
- Direct Ink Writing (DIW):
  ▪ Deposits droplets of drug solution onto a substrate to create thin films.
  ▪ Non-thermal, suitable for heat-sensitive drugs.
  ▪ Advantages: Precise control of drug loading and film thickness.
- Semi-Solid Extrusion (SSE):
  ▪ Extrudes paste-like materials for soft and flexible drug delivery devices.
  ▪ Advantages: Suitable for formulations that require room-temperature processing.

### 2.5. Fused Deposition Modeling (FDM)

FDM has gained considerable attention in pharmaceutical applications due to its simplicity, cost-effectiveness, and ability to process a wide range of thermoplastic polymers. FDM works by extruding a drug-loaded thermoplastic filament through a heated nozzle, which melts the material and deposits it layer by layer to form the desired object [24,35]. This method is particularly suitable for drug delivery systems, including patches and microneedles, as it allows for the incorporation of API directly into the filament during extrusion, resulting in uniform drug distribution [36]. Furthermore, the relatively low operational temperatures of FDM make it compatible with heat-sensitive APIs, which is critical in the formulation of many dermatological products [37].

FDM is used for fabricating personalized drug delivery systems using thermoplastic polymers such as Polylactic Acid (PLA), Polyvinyl Alcohol (PVA), and Polycaprolactone (PCL). A notable example is the fabrication of extended-release tablets loaded with ibuprofen, where the drug was incorporated into a polymeric filament, enabling precise control over the drug release profile [32]. Additionally, FDM has been employed in personalized transdermal patches, where hydrogel-based formulations are extruded to create layered structures that optimize drug diffusion through the skin. This technology has also been successfully applied in the development of buccal films designed for rapid drug absorption, particularly beneficial for pediatric and geriatric patients requiring non-invasive delivery routes [31].

FDM is cost-effective, making it ideal for small-scale production and research [26,38]. It enables rapid prototyping and customization, allowing for personalized patches and transdermal systems tailored to patients [24,35]. The ability to design complex geometries and control internal structures supports drug delivery systems with immediate or sustained release [36]. However, FDM has lower resolution than SLA, limiting its use in high-precision applications [39]. Additionally, it is restricted to thermoplastic polymers, which may not be suitable for all drug formulations [29].

FDM enhances yield and productivity in dermatological drug delivery by enabling rapid prototyping and tailored treatments. Its affordability and flexibility make it suitable for research and personalized applications [26]. For instance, FDM-printed transdermal patches conform to the patient’s skin, improving adhesion and localized drug delivery [24]. Additionally, FDM enables devices with controlled drug release, ensuring consistent therapeutic dosing [36].

The choice of filaments is crucial in FDM for dermatology. PLA, PVA, and PCL are preferred due to biocompatibility, strength, and low melting points, preventing API degradation [40,41]. PLA offers biodegradability and durability but is hydrophobic, limiting compatibility with hydrophilic drugs. PVA, being water-soluble, supports controlled drug release, while the flexibility and slow degradation of PCL make it ideal for wound dressings [30].

Conversely, some polymers are unsuitable for FDM. Chitosan lacks a melting point and thermally degrades, making it incompatible with high extrusion temperatures [42]. Similarly, the high viscosity and moisture sensitivity of PEO cause extrusion issues and inconsistent print quality, limiting its use [28]. These challenges highlight the importance of material selection to ensure efficient and reliable FDM-based dermatological devices.

While FDM improves productivity, customization, and drug delivery performance, careful filament selection is key to optimizing its application in dermatology. Balancing material properties and limitations ensures effective patient-specific treatments.

### 2.6. Stereolithography (SLA)

Stereolithography (SLA) is a 3D printing technique that uses a UV laser to cure a photosensitive resin layer by layer. SLA is known for its high-resolution capabilities, making it ideal for applications requiring intricate designs, such as microneedle arrays for drug delivery [23]. These microneedles, fabricated with photopolymerizable resins loaded with Active Pharmaceutical Ingredients (APIs), penetrate the stratum corneum, facilitating efficient drug absorption while eliminating the need for hypodermic injections [34].

SLA enhances resolution, precision, and structural integrity in 3D-printed dermatological devices. Its high resolution enables intricate designs, such as microneedle arrays, which improve drug penetration by bypassing the stratum corneum, enhancing therapeutic efficacy [25]. The SLA precision ensures consistent device geometry and optimized surfaces, leading to uniform drug release and effective skin contact. Additionally, its smooth surface finish minimizes discomfort, making it ideal for wearable drug delivery systems [29].

SLA efficiently produces high-quality prototypes and customized drug delivery devices, including personalized wound dressings. However, post-processing steps like resin cleaning and curing lower yield and extend manufacturing time, making it less suitable for large-scale production compared to FDM [39]. Despite this, its ability to create detailed, patient-friendly devices makes it invaluable for precision-driven dermatological applications.

The suitability of SLA resins in dermatology depends on biocompatibility and curing properties. PEGDA and polyurethane-based resins are preferred for their non-toxic nature, flexibility, and durability. The PEGDA hydrophilicity supports controlled drug release, making it ideal for skin-contact applications [23]. In contrast, rigid or low-biocompatibility resins can cause skin irritation and reduce flexibility, limiting their use in wearable patches or microneedles. Additionally, poorly curing photosensitive resins can lead to incomplete polymerization, compromising mechanical properties and safety.

Thus, the SLA high precision and smooth finishes enhance dermatological 3D-printed devices. However, resin selection and post-processing considerations are crucial to optimizing performance while addressing its limitations.

### 2.7. Selective Laser Sintering (SLS)

Selective Laser Sintering (SLS) is a 3D printing method that uses a laser to fuse powdered material layer by layer. SLS is known for its ability to create porous structures, which can be advantageous for drug delivery devices that require a high surface area for drug loading [26]. This method has been explored for orodispersible tablets containing poorly water-soluble drugs, enhancing their bioavailability through porous structures that facilitate rapid dissolution in saliva [28]. Additionally, SLS has been applied in implantable drug delivery devices, such as biodegradable scaffolds loaded with antibiotics, providing localized drug release for post-surgical infection prevention [31].

SLS offers the benefit of producing porous structures that enhance drug loading and release, making it useful for transdermal patches and wound dressings [28]. Another advantage of SLS is that it does not require support structures during printing, which simplifies the manufacturing process [23]. However, the cost of SLS printers and materials is relatively high, making it less feasible for routine pharmaceutical applications [29]. Furthermore, the need for extensive post-processing to remove excess powder can be time-consuming and may affect the final product’s quality [26].

Materials suitable for SLS in dermatological applications include thermoplastic polymers such as Polycaprolactone (PCL) and Thermoplastic Polyurethanes (TPUs). These materials exhibit excellent flexibility, biocompatibility, and durability, essential for wearable and conformable devices. For instance, the PCL slow degradation rate makes it ideal for long-term applications like wound healing scaffolds, while the TPU elasticity allows to produce comfortable patches [41]. Conversely, materials with high brittleness or poor thermal stability, such as chitosan or some rigid resins, are less suitable due to challenges in maintaining structural integrity during the sintering process or in the final product [42]. The choice of material greatly influences both the yield and functional effectiveness of SLS-printed dermatological devices, necessitating careful selection to optimize performance.

### 2.8. Direct Ink Writing (DIW)

Direct Ink Writing (DIW) is another technique used in pharmaceutical applications, particularly for creating thin films and drug-loaded patches. It involves depositing droplets of drug solution onto a substrate to form a film [43]. It has gained attention for its ability to deposit precise microdoses of drugs, making it highly suitable for personalized medicine and multidrug combination therapies. This technique has been successfully used to create layered polypill formulations, where multiple APIs with different release kinetics are printed in a single tablet, allowing for controlled and sequential drug release [33].

Direct Ink Writing (DIW) influences the uniformity and precision of drug delivery devices, particularly for dermatological preparations requiring controlled dosing and thin-film applications. By allowing for the precise deposition of drug solutions, this method enhances the accuracy of drug distribution within patches, ensuring consistent therapeutic outcomes. For instance, personalized patches for localized treatment of skin conditions can be produced with minimal wastage of active pharmaceutical ingredients [43]. Additionally, the ability to integrate multiple drugs into a single layer opens avenues for combination therapies tailored to complex dermatological conditions [29].

The method is most suitable for low-viscosity liquid formulations that can flow smoothly through nozzles, such as solutions or emulsions containing hydrophilic polymers like Polyvinyl Alcohol (PVA). These materials enable the creation of uniform and stable films with excellent adhesion to substrates. However, inkjet printing is not ideal for high-viscosity or particulate-laden formulations, as they may clog the nozzle or produce inconsistent layers [23]. This limitation underscores the need to optimize formulation properties for effective use in inkjet-based dermatological applications. The precision and adaptability of inkjet printing make it a valuable tool for creating advanced, patient-specific treatments in dermatology.

### 2.9. Why Fused Deposition Modeling (FDM) Stands out for Dermatological Applications

Fused Deposition Modeling stands out as a preferred method for printing dermatological preparations due to its versatility, cost-effectiveness, and ability to process biocompatible polymers suitable for drug delivery. In contrast to SLA and SLS, which require expensive equipment and post-processing, FDM offers a simpler and more economical solution for fabricating drug-loaded patches. Additionally, the FDM compatibility with a wide range of thermoplastic polymers allows for the development of customized drug delivery systems tailored to specific patient needs [24].

The ability to incorporate APIs directly into the filament during extrusion is a key advantage of FDM over other methods, as it ensures homogeneous drug distribution and reduces the risk of dose variability [36]. Furthermore, the low operational temperatures of FDM make it suitable for processing heat-sensitive drugs, which is a limitation for many other 3D printing methods [37]. Overall, the FDM combination of flexibility, accessibility, and compatibility with pharmaceutical-grade materials makes it an ideal choice for advancing dermatological therapies.

### 2.10. Post-Processing in Three-Dimensional (3D) Printing

Post-processing is a crucial step to refine the mechanical, structural, and biological properties of 3D-printed materials, ensuring their suitability for medical use. Various post-processing techniques are employed across different 3D printing methods, including Fused Deposition Modeling (FDM), Stereolithography (SLA), Selective Laser Sintering (SLS), and inkjet printing, to enhance surface finish, improve drug release profiles, and optimize mechanical strength.

In FDM, the layer-by-layer deposition process results in rough surfaces and potential interlayer delamination, which can affect drug release kinetics and mechanical stability. Solvent vapor smoothing is a key post-processing method used to improve surface uniformity by partially dissolving polymer layers, creating a smoother finish and enhancing the controlled release of Active Pharmaceutical Ingredients (APIs) [1]. Annealing is another approach, where the printed structure is heated to below its melting point, reducing residual stresses and increasing mechanical integrity [44].

SLA-printed objects require extensive post-curing using UV light to achieve complete polymerization and enhance crosslinking, improving mechanical strength and drug stability [31]. Additionally, chemical post-processing techniques, such as ethanol or isopropanol rinsing, are employed to remove residual uncured resin, which can be cytotoxic if not properly eliminated [44]. These post-processing steps are essential to ensure SLA-printed materials are safe and effective for biomedical applications.

SLS utilizes a laser to sinter powdered polymers layer by layer, resulting in porous structures that can be advantageous for implantable drug delivery devices. However, the powder-based process requires extensive post-processing to remove unsintered particles, which may impact the overall drug content and uniformity. Heat treatment is often applied to enhance the mechanical properties of sintered structures, reducing brittleness and ensuring long-term durability [44]. Furthermore, surface coating with biocompatible polymers can be utilized to modify drug release kinetics and improve biocompatibility [31].

Inkjet-based 3D printing is primarily used for fabricating personalized drug formulations, offering precise control over dosage and drug layering. However, inkjet-printed structures often require thermal post-processing to stabilize drug-polymer matrices, preventing degradation during storage. Additionally, drying and solvent evaporation techniques help improve mechanical stability and prevent drug crystallization, ensuring consistent drug release profiles [34].

Post-processing plays a vital role in optimizing the performance and safety of 3D-printed biomedical devices. Techniques such as solvent vapor smoothing, UV curing, heat treatment, and chemical post-processing are essential for enhancing mechanical properties, drug release profiles, and biocompatibility. As 3D printing continues to evolve, integrating advanced post-processing strategies will be crucial in overcoming current limitations and expanding the applications of personalized drug delivery and tissue engineering [1].

### 2.11. Comparison of Fused Deposition Modeling (FDM) with Other Three-Dimensional (3D) Printing Technologies

Fused Deposition Modeling (FDM) offers distinct advantages in dermatological drug delivery, primarily due to its affordability, simplicity, and compatibility with various thermoplastic polymers such as PLA, PVA, and PCL [24]. It allows for the straightforward and cost-effective production of customized drug-loaded devices, ideal for personalized patches and transdermal systems. However, FDM typically provides lower resolution compared to other techniques, restricting its suitability for applications requiring precise features, such as microneedles [39].

In comparison, Stereolithography (SLA) delivers superior resolution and surface quality, making it suitable for intricate applications, including microneedle arrays and detailed skin patches [25]. However, the SLA necessity for UV-curable resins limits material choices and increases production complexity due to additional post-curing processes. These factors may affect drug stability and bioactivity due to prolonged exposure to UV light [23].

Selective Laser Sintering (SLS) stands out due to its powder-based approach, offering design freedom and eliminating the need for supports. Additionally, it is advantageous for fabricating porous drug delivery systems and complex internal structures [29]. However, the post-processing required to remove excess powder can be cumbersome, affecting drug yield and process efficiency. Additionally, the thermal conditions involved in SLS may limit its suitability for thermolabile Active Pharmaceutical Ingredients (APIs).

Direct Ink Writing (DIW) differs from FDM by allowing for the use of viscous, paste-like materials and bio-inks, enabling the fabrication of highly porous and bioactive structures that are suitable for regenerative applications such as wound dressings and tissue scaffolds [34]. Nonetheless, the relatively slower printing speed and complexity of DIW in formulation adjustments restrict its practicality for large-scale or rapid prototyping scenarios.

In conclusion, while FDM is highly beneficial for cost-effective, customizable, and straightforward dermatological applications, each 3D printing technology offers unique strengths and limitations. The table below (Table 2) illustrates the comparison of various 3D printing technologies. Selecting the appropriate method thus requires careful consideration of the specific dermatological application, desired resolution, material properties, and production scalability.

## 3. Problem Statement

There is currently a lack of comprehensive review articles focused specifically on the selection of filaments for Fused Deposition Modeling (FDM) 3D printing in dermatological preparations leading to challenges in choosing the appropriate filament causing increased trial-and-error, wasted resources, and potential failures in developing effective drug delivery systems. Hence, this review aims to bridge this gap by providing a detailed analysis of the available filaments, their properties, and suitability for 3D printing of preparations for dermatological applications.

## 4. Polymers Used for Three-Dimensional (3D) Printing Fused Deposition Modeling (FDM)

### 4.1. List of Polymers

Polyethylene Glycol (PEG)
Kolliphor P188 (Poloxamer 188)
Polyvinylpyrrolidone (Kollidon 12PF)
Vinylpyrrolidone vinyl acetate copolymer (Kollidon VA64)
Polylactic Acid (PLA)
Polyvinyl Alcohol (PVA)
Chitosan
Hydroxypropyl Cellulose (HPC)
Polycaprolactone (PCL)
Thermoplastic Polyurethanes (TPUs)
Eudragit (methyl prop-2-enoate;2-methylprop-2-enoic acid)
Ethylene Vinyl Acetate (EVA)

### 4.2. Filaments Specific to Dermatological Preparations

PVA and PEG: Ideal for creating drug delivery devices requiring controlled dissolution and flexibility.

PCL and TPU: Used for wound dressings and wearable patches due to their flexibility and prolonged degradation properties.

Eudragit: Suitable for pH-sensitive and sustained release systems for localized drug delivery.

EVA: Employed in transdermal systems for its sustained drug release capability.

HPC and Kollidon: Preferred for their biocompatibility and excellent drug release properties.

### 4.3. Properties of Polymers

#### 4.3.1. Polyethylene Glycol (PEG)

**Figure 1:**
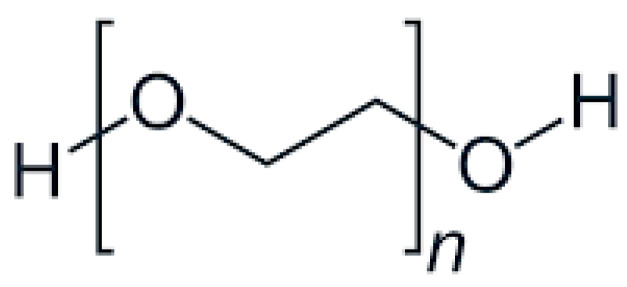
Chemical structure of PEG.

PEG is commonly used as a plasticizer in FDM due to its excellent hydrophilicity and flexibility-enhancing properties. Its structure, characterized by repeating ethylene oxide units [45], significantly lowers extrusion temperatures and improves filament flexibility, as illustrated in Figure 1.

General formula: HO-(CH_2_CH_2_O)_n_-H
Functional Groups:

Ether Groups (-O-):

Provide hydrophilicity through hydrogen bonding with water molecules, enhancing solubility.

Offer chemical inertness, allowing for compatibility with APIs.

Terminal Hydroxyl Groups (-OH):

Increase hydrophilicity by forming hydrogen bonds.

Enable conjugation with APIs, improving solubility and stability [46].

Physical Description:
  - Low molecular weight PEGs: Colorless, viscous liquids.
  - High molecular weight PEGs: White, waxy solids [46,47].
  - Odorless [46,47].
  - Solubility: Highly soluble in water; soluble in organic solvents such as acetone, ethanol, and chloroform [46].

Impact on Stratum Corneum Permeation:
  - PEG increases hydration of the stratum corneum and interacts with skin lipids, facilitating the delivery of hydrophilic APIs.

##### Properties of Polyethylene Glycol (PEG)

In Fused Deposition Modeling (FDM), PEG is often employed as a plasticizer to improve filament flexibility and reduce extrusion temperatures [48]. Different molecular weights of PEG (e.g., 4000, 6000) are used to modify the mechanical properties of the filaments. The crystallinity and molecular configuration (helix or trans planar zigzag) of PEO greatly influence its properties and application [49]. In this study carried out by Azizoğlu & Özer [27], PEG was added to polymer blends to reduce the extrusion temperature and improve the flexibility of the filaments, which helped make the resulting filaments more flexible and easier to handle during 3D printing. 

PEG has good biocompatibility, which makes it a preferred choice in biomedical and pharmaceutical applications [50,51,52]. Its non-toxic nature ensures safety in dermatological preparations, making PEG particularly valuable for use in 3D-printed drug delivery systems, such as transdermal patches and wound dressings.

PEG is a hydrophilic polymer with high solubility in water and various organic solvents. The hydrophilicity of PEG can pose challenges in FDM, as it may absorb moisture during printing, which can lead to filament degradation, bubbling, or poor layer adhesion during the extrusion process [53].

The melting point of PEG depends on its molecular weight. For example, PEG 4000 and PEG 6000 have melting points in the range of 50–60 °C [27]. These relatively low melting points can be beneficial in FDM, as they reduce the extrusion temperature required, making PEG ideal for blending with other polymers to create composite filaments. The reduced extrusion temperature also allows for the incorporation of heat-sensitive APIs without significant thermal degradation [27].

The PEG viscosity varies based on its molecular weight, impacting the ease of extrusion during 3D printing. Low molecular weight PEGs, such as PEG 4000, exhibit lower viscosity, which can enhance flow properties and facilitate smoother extrusion. High viscosity PEGs, such as PEG 8000 on the other hand, can lead to nozzle clogging or inconsistent extrusion, posing challenges in the printing process [54]. Spreadability, which influences how well the material layers adhere to one another, is also affected by viscosity. The PEG ability to act as a plasticizer improves the spreadability of the filament, resulting in better layer adhesion and a more uniform surface finish.

##### Advantages and Disadvantages of Polyethylene Glycol (PEG) in Fused Deposition Modeling (FDM)

PEG offers several benefits when used in FDM. One of its primary advantages is its plasticizing effect, which improves the flexibility of FDM filaments, reduces brittleness, and lowers extrusion temperatures. This makes PEG particularly useful for enhancing the printability of rigid polymers such as PLA and PCL, making them more suitable for drug delivery applications that require pliable materials [27]. Additionally, the PEG biocompatibility ensures that it is safe for use in dermatological applications where prolonged skin contact is necessary. The relatively low extrusion temperatures of PEG-containing filaments make it compatible with various heat-sensitive APIs, ensuring that the therapeutic efficacy of the drug is not compromised during printing [55].

However, there are also significant drawbacks to using PEG in FDM. One major limitation is its moisture sensitivity. PEG is highly hygroscopic, meaning it readily absorbs moisture from the environment which can lead to issues during the printing process, such as filament swelling, bubbling, and poor adhesion between layers, ultimately affecting the quality of the printed object. Additionally, PEG-based filaments may lack the mechanical strength required for certain applications. Pure PEG or high PEG content blends tend to be softer and less durable, which may limit their use in structural components of drug delivery devices [28].

While PEG has several properties that make it attractive for FDM, including its plasticizing effect and biocompatibility, it is not ideally suited for use as a primary polymer in FDM. The hygroscopic nature of PEG poses significant challenges during the printing process, as moisture absorption can lead to poor print quality and inconsistent mechanical properties. Additionally, the PEG lack of mechanical strength limits its applicability in creating robust structures that are required for certain drug delivery devices.

Instead, PEG is better suited as an additive or plasticizer in combination with other polymers to enhance their flexibility and reduce extrusion temperatures. For instance, PEG can be blended with PLA or PCL to improve the overall flexibility and printability of the filament, making it more suitable for applications that require soft and conformable drug delivery systems [27].

#### 4.3.2. Kolliphor P188 (Poloxamer 188)

**Figure 2:**
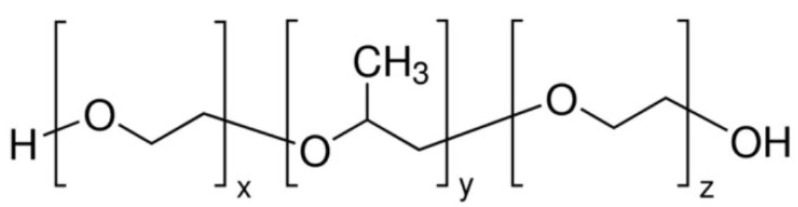
Chemical structure of Kolliphor P188.

Kolliphor P188 is an amphiphilic polymer known for enhancing drug permeation and filament flexibility in topical drug delivery systems. Its unique molecular structure supports better integration with hydrophobic and hydrophilic drug compounds, as depicted in Figure 2.

General Formula: HO–(C_2_H_4_O)_n_–(C_3_H_6_O)_m_–(C_2_H_4_O)_n_–H

Functional Groups:

Polyoxyethylene (PEO) Chains: Hydrophilic segments that enhance water solubility.

Polyoxypropylene (PPO) Chains: Hydrophobic segments that facilitate interaction with hydrophobic drug molecules.

Physical Description:

Appearance: Solid, white to slightly yellowish, coarse-grained powder with a waxy consistency. [56,57]

Odor: Faint, characteristic odor.

- Melting Point: Approximately 52 °C (125 °F). [56]
- Boiling Point: N/A (decomposes before boiling).
- Solubility: Freely soluble in water and ethanol; insoluble in diethyl ether, paraffin, and fatty oils. [58]

Impact on Stratum Corneum Permeation:
  - The amphiphilic nature of Kolliphor P188 allows it to interact with both hydrophilic and hydrophobic regions of the stratum corneum, enhancing the permeation of Active Pharmaceutical Ingredients (APIs) through the skin.

##### Properties of Kolliphor P188

Kolliphor P188 is well-known for its excellent biocompatibility, which makes it suitable for use in a wide range of pharmaceutical and biomedical applications. This polymer is non-toxic and non-irritant, making it particularly suitable for topical applications where direct contact with the skin is necessary. Its biocompatibility ensures that formulations containing Kolliphor P188 can be used safely for dermatological treatments, minimizing the risk of skin irritation or adverse reactions.

Kolliphor P188 is highly soluble in water, which is advantageous for applications requiring the homogeneous distribution of active ingredients. However, its high solubility also means that it can absorb moisture from the environment, potentially leading to issues such as poor adhesion between layers during the FDM process, which may affect the mechanical properties of the printed object.

The melting point of Kolliphor P188 is relatively low, ranging from 52 °C to 57 °C, which is beneficial for FDM as it allows for lower extrusion temperatures [27]. This lower melting point is particularly useful for incorporating heat-sensitive APIs without causing thermal degradation. However, at certain concentrations and temperatures, Kolliphor P188 may lead to brittleness or fluidity issues, which can limit its application in filament formulations [27]. Careful optimization of the polymer concentration is needed to avoid these issues and to maintain suitable mechanical properties for FDM.

Kolliphor P188 exhibits relatively low viscosity compared to other polymers, which contributes to its ability to reduce the extrusion temperature during FDM. The low viscosity ensures that the material can be extruded smoothly, reducing the likelihood of nozzle clogging. However, the low viscosity can also result in reduced mechanical strength of the printed parts, which may limit their suitability for certain applications requiring robust structures. The spreadability of Kolliphor P188 also influences the quality of the printed layers, as its plasticizing effect improves the layer adhesion and surface uniformity, leading to better-quality 3D-printed products.

##### Advantages and Disadvantages of Using Kolliphor P188 in Fused Deposition Modeling (FDM)

Kolliphor P188 provides several advantages in FDM-based drug delivery systems. Its plasticizing effect enhances flexibility and reduces brittleness in rigid polymers, making it ideal for stable, flexible filaments [27]. Its low melting point allows for lower extrusion temperatures, benefiting heat-sensitive APIs. Additionally, its biocompatibility minimizes the risk of skin irritation, making it a safe option for dermatological formulations [55].

However, Kolliphor P188 has limitations. Its high solubility and hygroscopic nature can lead to moisture absorption, causing poor adhesion and reduced mechanical strength in printed objects. At certain concentrations, it can result in brittleness or excessive fluidity, requiring careful polymer optimization for ideal mechanical and printing properties [27].

Compared to PLA, PEG, and PEO, Kolliphor P188 has distinct advantages and challenges. PLA, widely used for biomedical applications, often requires plasticizers to improve flexibility. Blending Kolliphor P188 with PLA enhances flexibility and reduces brittleness, making PLA-based filaments more suitable for dermatological use [27]. Similarly, while PEG serves as a plasticizer in FDM, the lower melting point of Kolliphor P188 makes it preferable for heat-sensitive APIs, though moisture sensitivity remains a drawback.

Due to its hygroscopic nature and brittleness at certain concentrations, Kolliphor P188 is best used as a plasticizer rather than a primary polymer. Blending it with PLA or PCL improves printability and mechanical properties, making it ideal for dermatological drug delivery applications [27].

Given the limitations of Kolliphor P188 in FDM, other 3D printing methods may be more suitable for this polymer. Inkjet printing and Stereolithography (SLA) are potentially better options for Kolliphor P188-based formulations. Inkjet printing is a non-thermal process, which makes it ideal for heat-sensitive polymers like Kolliphor P188 [43]. SLA, which uses photopolymerizable resins, can also be adapted to incorporate Kolliphor P188 as part of a resin mixture, allowing for the creation of hydrogel-based structures with high precision and controlled drug release [23].

#### 4.3.3. Polyvinylpyrrolidone (Kollidon 12PF)

**Figure 3:**
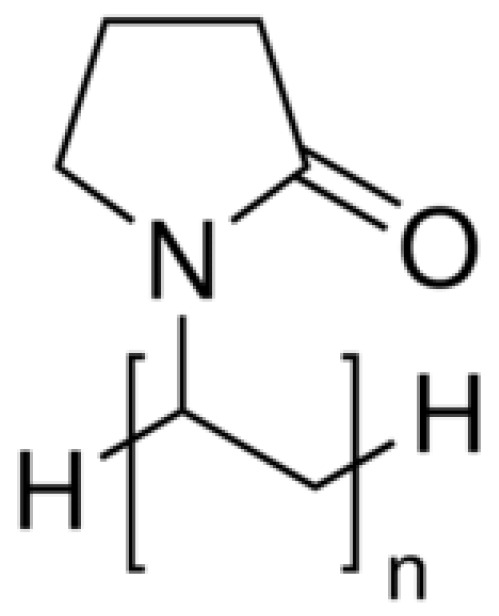
Chemical structure of Kollidon 12PF.

Kollidon 12PF, a polyvinylpyrrolidone-based polymer, is widely valued for its biocompatibility and strong drug-dispersing capabilities. The polymer’s molecular architecture, highlighted in Figure 3, promotes uniform drug distribution within printed formulations.

General/Molecular Formula: (C_6_H_9_NO)_n_
Functional Groups:
  - Lactam Group (-C=O and -NH):
    ▪ Contributes to hydrophilicity by forming hydrogen bonds with water and APIs.
    ▪ Aids in API solubilization and stabilization.
  - Hydrocarbon Backbone:
    ▪ Provides moderate hydrophobicity, aiding in the interaction with non-polar APIs.

Physical Description:
  - Appearance: White, spray-dried powder.
  - Odor: Faint, characteristic odor.
  - Boiling Point: N/A (Decomposes before boiling).
  - Solubility: Highly soluble in water and a variety of organic solvents, including ethanol, glycerin, and methanol. Insoluble in non-polar solvents like cyclohexane and toluene [59].

Impact on Stratum Corneum Permeation:
  - The hydrophilic lactam groups facilitate hydration of the stratum corneum, increasing permeability.
  - Enhances the compatibility and stability of APIs for topical and transdermal applications.

##### Properties of Kollidon 12PF

Kollidon 12PF is a biocompatible polymer, making it suitable for use in pharmaceutical and biomedical applications where direct contact with biological tissues is required. Its non-toxic nature ensures safety for dermatological applications, such as transdermal patches and wound dressings. The biocompatibility of Kollidon 12PF plays an essential role in ensuring that it does not cause any adverse skin reactions, which is a crucial factor in dermatological preparations. This property makes Kollidon 12PF a reliable option for use in drug delivery systems that need to be in direct contact with the skin [55]

Kollidon 12PF is highly soluble in water, which makes it an excellent candidate for formulations requiring controlled release or enhanced drug solubility. The high solubility of Kollidon 12PF allows it to form homogenous mixtures with APIs, which improves drug dispersion and enhances bioavailability. However, this high solubility may also lead to challenges during the printing process, as the hygroscopic nature of Kollidon 12PF can result in moisture absorption, potentially leading to filament degradation and inconsistent printing quality [55].

Kollidon 12PF has a glass transition temperature (Tg) of approximately 90 °C, which means it requires relatively high processing temperatures for extrusion [27]. This high Tg contributes to the structural stability of the printed object, providing good mechanical strength and rigidity. However, the high processing temperatures required to extrude Kollidon 12PF may limit its suitability for temperature-sensitive APIs, as they could degrade during the extrusion process. To overcome this limitation, plasticizers are often added to reduce the extrusion temperature, thereby preserving the integrity of the active compounds [55].

The viscosity of Kollidon 12PF is relatively low, which helps improve its flow properties during extrusion. The polymer’s low viscosity facilitates smoother extrusion, resulting in fewer issues such as nozzle clogging during the 3D printing process. Additionally, the ability of Kollidon 12PF to act as a matrix polymer helps improve the spreadability of the filament, resulting in better adhesion between layers and more consistent print quality [55]. The enhanced spreadability of Kollidon 12PF helps achieve uniform surface finishes in the final printed object, making it ideal for producing drug delivery devices for dermatological applications.

##### Advantages and Disadvantages of Kollidon 12PF in Fused Deposition Modeling (FDM)

One of the primary advantages of Kollidon 12PF in FDM is its excellent solubility, which allows for the homogeneous distribution of APIs in the filament. This results in improved drug release profiles, making it a suitable choice for creating 3D-printed transdermal patches and other topical drug delivery systems [27]. Another significant advantage is its biocompatibility, which ensures that the printed products are safe for use in contact with the skin. Additionally, the ability of Kollidon 12PF to form solid dispersions with APIs helps improve the bioavailability of poorly soluble drugs, making it a valuable polymer for drug delivery applications [55].

However, there are also limitations to using Kollidon 12PF in FDM. The high glass transition temperature means that the polymer requires high processing temperatures for extrusion, which may not be suitable for temperature-sensitive compounds [55]. While plasticizers can be added to lower the extrusion temperature, they may also compromise the mechanical properties of the printed object, potentially leading to reduced stability. Moreover, the hygroscopic nature of Kollidon 12PF can lead to moisture absorption, which may affect the quality of the filament and the final printed product.

Kollidon 12PF has several properties that make it suitable for use in FDM, including its excellent solubility, biocompatibility, and ability to enhance drug bioavailability. However, its high glass transition temperature and hygroscopic nature present challenges during the printing process. The high processing temperatures required for Kollidon 12PF may limit its use with temperature-sensitive APIs, and its moisture absorption can lead to filament degradation and inconsistent print quality. Therefore, Kollidon 12PF is best used in combination with plasticizers or other polymers to reduce extrusion temperatures and enhance printability [55].

Hot-melt extrusion combined with FDM remains a viable approach for producing drug-loaded filaments, especially when plasticizers are used to lower the extrusion temperature. However, inkjet printing and Stereolithography (SLA) could also be considered for Kollidon 12PF-based formulations. Inkjet printing is a non-thermal process, which makes it ideal for heat-sensitive APIs [43]. SLA, which uses photopolymerizable resins, can also be adapted to incorporate Kollidon 12PF, allowing for high-precision structures with controlled drug release properties [23].

#### 4.3.4. Vinylpyrrolidone Vinyl Acetate Copolymer (Kollidon VA64)

**Figure 4:**
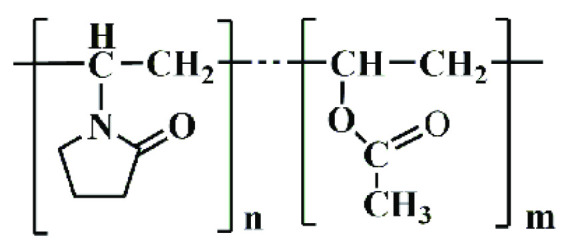
Chemical structure of Kollidon VA64.

Kollidon VA64 combines hydrophilic and hydrophobic characteristics, enhancing drug solubility and bioavailability in dermatological preparations. Its molecular structure facilitates the formation of stable, flexible films suitable for transdermal drug delivery, as shown in Figure 4.

General Formula: (C_6_H_9_NO)_n_(C_4_H_6_O_2_)_m_
Composition: Approximately 60% N-vinylpyrrolidone and 40% vinyl acetate by weight (BASF, 2022).

Functional Groups:
  - Lactam Group (-C=O and -NH):
    ▪ Contributes to hydrophilicity and compatibility with APIs.
    ▪ Forms hydrogen bonds, enhancing solubility and drug dispersion.

- Acetate Ester Group (-COO-):
  ▪ Adds hydrophobicity, improving the copolymer’s ability to interact with hydrophobic APIs.
  ▪ Enhances film-forming properties for coatings.

Physical Description:
  - Appearance: White to slightly yellowish spray-dried powder.
  - Odor: Faint characteristic odor.
  - Melting Point: Amorphous; no defined melting point.
  - Glass Transition Temperature: 101 °C.
  - Solubility: Soluble in water, alcohols (ethanol, isopropanol), and organic solvents like methylene chloride. Insoluble in non-polar solvents such as cyclohexane (BASF, 2022).

Impact on Stratum Corneum Permeation:
  - Hydrophilic and hydrophobic segments in the copolymer facilitate drug dispersion and enhance permeation through the lipid-rich stratum corneum.
  - Forms stable films, improving the controlled release and skin adhesion of dermatological formulations.

##### Properties of Kollidon VA64

Kollidon VA64 is a biocompatible polymer, which is a crucial property for dermatological preparations where direct contact with the skin is required. The non-toxic nature of Kollidon VA64 ensures that it is safe for use in drug delivery systems, minimizing the risk of skin irritation or adverse reactions. This biocompatibility is especially important for 3D-printed transdermal patches and other dermatological devices that need to be in prolonged contact with the skin.

Kollidon VA64 and Kollidon VA64 Fine are soluble in both water and alcohols, which makes them highly suitable for a range of applications requiring controlled release or enhanced drug solubility. The high solubility of Kollidon VA64 allows it to form homogeneous mixtures with APIs, thereby improving drug dispersion and enhancing bioavailability. This characteristic is particularly beneficial for FDM 3D printing, as it ensures that the active ingredients are uniformly distributed throughout the printed structure. However, the hygroscopic nature of Kollidon VA64 can pose challenges during the printing process, as it may absorb moisture from the environment, potentially leading to filament degradation and inconsistent printing quality [55].

Kollidon VA64 has a Tg of approximately 101 °C, and a high temperature resistance up to 220 °C, which contributes to the structural stability and rigidity of the printed object. This high Tg, however, means that relatively high processing temperatures are required for extrusion, which may limit its use with temperature-sensitive APIs that could degrade during the extrusion process. To address this limitation, plasticizers like PEG are often added to reduce the extrusion temperature, thereby preserving the integrity of the active compounds while maintaining the mechanical properties of the filament [27].

Kollidon VA64 exhibits good viscosity characteristics, which facilitate its smooth extrusion during the FDM process. Its viscosity helps in reducing nozzle clogging, resulting in consistent printing and smooth surface finishes. Solutions of Kollidon VA64 exhibit good flow behavior, enabling easy extrusion with fewer printing defects. Additionally, the excellent film-forming properties of Kollidon VA64 ensure that printed layers adhere well to each other, which is critical for achieving robust and durable printed structures. These attributes make Kollidon VA64 particularly suitable for applications that require uniform drug delivery and reliable mechanical strength [55].

Kollidon VA64 absorbs only about one-third of the amount of water absorbed by povidone, which reduces its hygroscopicity compared to other similar polymers. This property makes Kollidon VA64 less prone to moisture-related issues during storage and processing. However, some level of moisture absorption can still occur, which requires careful handling to prevent adverse effects on filament stability and print quality.

##### Advantages and Disadvantages of Kollidon VA64 in Fused Deposition Modeling (FDM)

Kollidon VA64 is biocompatible, making it a safe option for dermatological applications, such as transdermal patches and topical formulations [55]. However, its high glass transition temperature (Tg) requires elevated extrusion temperatures, which may not be suitable for temperature-sensitive APIs. While plasticizers can lower extrusion temperatures, they may also alter mechanical stability [60]. Additionally, the hygroscopic nature of Kollidon VA64 makes it prone to moisture absorption, potentially affecting filament quality and print stability if not properly managed [55].

Compared to PLA and PEG, Kollidon VA64 offers better solubility and API compatibility. While PLA is widely used for its biodegradability and mechanical strength, it often requires plasticizers for flexibility. In contrast, Kollidon VA64 provides superior solubility, making it more suitable for drug delivery applications requiring enhanced bioavailability [55].

PEG is often used as a plasticizer in FDM to improve filament flexibility. While Kollidon VA64 also requires plasticizers for lower temperature processing, it offers better structural stability and mechanical strength than PEG and PEO, making it ideal for applications needing rigidity and durability. Blending Kollidon VA64 with plasticizers or other polymers further enhances flexibility, expanding its use in various 3D printing applications.

Despite its advantages, the high Tg and hygroscopic nature of Kollidon VA64 pose challenges in FDM-based formulations. The high extrusion temperature requirement may limit its use with heat-sensitive APIs, while moisture absorption can degrade filament stability. Thus, Kollidon VA64 is best combined with plasticizers or other polymers to reduce extrusion temperatures and enhance printability [55]. While FDM is a viable option for Kollidon VA64, other 3D printing methods may be more suitable in certain scenarios. For example, inkjet printing and Stereolithography (SLA) could also be considered for Kollidon VA64-based formulations. Inkjet printing is a non-thermal process, which makes it ideal for heat-sensitive APIs, while SLA can create high-precision structures using photopolymerizable resins, which can be adapted to incorporate Kollidon VA64 for enhanced drug delivery properties [55].

#### 4.3.5. Polylactic Acid (PLA)

**Figure 5:**
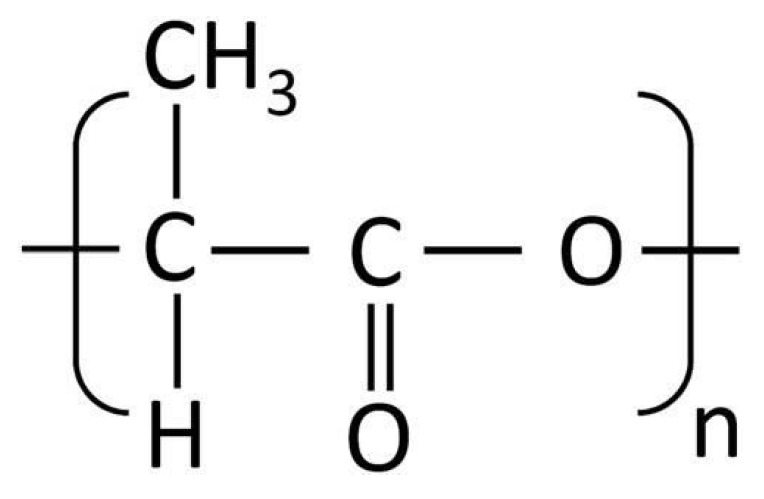
Chemical structure of PLA.

PLA is a biodegradable thermoplastic commonly utilized in FDM due to its mechanical robustness and environmental sustainability. Its molecular structure, consisting of repeating lactic acid units, provides structural integrity, as illustrated in Figure 5. Although rigid, it often requires plasticizers to enhance flexibility for dermatological uses.

General Formula: (C_3_H_4_O_2_)_n_
Functional Groups:
  - Ester Group (-COO-): Provides hydrophobicity, reducing water solubility and enhancing compatibility with hydrophobic APIs [61].
  - Hydroxyl Group (-OH, as terminal group): Adds limited hydrophilicity, allowing for minor interaction with hydrophilic APIs.

Physical Description:
  - Appearance: Transparent to opaque, shiny, and brittle.
  - Odor: Odorless.
  - Melting Point: 140–210 °C, depending on crystallinity. [62,63]
  - Glass Transition Temperature: 50–75 °C.
  - Solubility: Soluble in organic solvents such as chloroform and benzene; insoluble in water [61,63].

Impact on Stratum Corneum Permeation:
  The hydrophobic nature of PLA enables controlled release of encapsulated drugs through the lipid-rich stratum corneum.
  Provides structural integrity in formulations, making it suitable for transdermal patches and drug-loaded microneedles [61].

##### Properties of Polylactic Acid (PLA)

PLA is considered to be biocompatible, which makes it a suitable candidate for drug delivery systems that come into direct contact with the skin. This property is particularly important for dermatological preparations, where polymers must be safe for prolonged contact with human tissues. The degradation products of PLA are lactic acid monomers, which are naturally metabolized by the body, reducing the likelihood of adverse reactions. The biocompatibility of PLA ensures that it can be safely used in a wide range of biomedical applications, including wound healing patches and transdermal drug delivery systems [24].

PLA is hydrophobic, which impacts its ability to incorporate and release hydrophilic drugs effectively. This hydrophobic nature limits the drug-loading capacity for certain types of drugs, particularly those that are water-soluble. This presents a significant challenge for the use of PLA in dermatological preparations that require controlled release of hydrophilic APIs. Strategies to overcome this include the use of plasticizers or blending PLA with other polymers that possess better hydrophilic characteristics. Recent studies have demonstrated the effectiveness of such modifications in improving the drug-loading capacity and release profiles of PLA-based formulations [30].

PLA has a melting point in the range of 150–180 °C and a glass transition temperature (Tg) of about 55 °C, which are well within the range for processing using FDM technology. The moderate melting point of PLA allows for efficient extrusion through the 3D printer nozzle without the need for extremely high temperatures, which could be detrimental to the stability of active pharmaceutical ingredients. However, its relatively low Tg means that PLA parts can deform under elevated temperatures, which can be a limiting factor in the production of structures that need to maintain their form in warm environments [55].

The viscosity and rheological behavior of PLA are suitable for FDM, allowing for consistent extrusion and good layer adhesion during the printing process. The viscosity of PLA is impacted by the molecular weight, which must be carefully controlled to ensure optimal print quality. The good spreadability of PLA facilitates the deposition of smooth, consistent layers during the FDM process, which is crucial for the production of high-quality, functional parts. However, the addition of plasticizers or blending with other polymers can further improve the spreadability and printability of PLA filaments, making them more suitable for intricate drug delivery applications [24].

##### Advantages and Disadvantages of Using Polylactic Acid (PLA) in Fused Deposition Modeling (FDM)

One of the main advantages of PLA in FDM is its ease of processing. PLA filaments are readily available, and their processing does not require extremely high temperatures, making them user-friendly and suitable for a wide range of 3D printers [64]. Additionally, PLA is biodegradable, which is a significant advantage for dermatological applications where biodegradability can enhance safety and environmental sustainability [55]. The ability of PLA to degrade into lactic acid makes it particularly appealing for short-term use medical devices, such as wound dressings and drug delivery systems.

However, there are also some drawbacks to using PLA for dermatological preparations. The hydrophobic nature of PLA makes it less suitable for loading hydrophilic drugs, which can result in limited drug release from the printed structure. Moreover, the brittleness of PLA can lead to mechanical failure, especially in applications where flexibility is required. Studies have shown that incorporating plasticizers like PEG or blending PLA with more flexible polymers can help to overcome these issues, improving both the mechanical properties and drug delivery characteristics of PLA-based filaments [30].

Compared to other commonly used FDM polymers like Polyethylene Glycol (PEG), Polyvinyl Alcohol (PVA), and Polycaprolactone (PCL), PLA stands out for its ease of printing and biodegradability. PCL, for instance, has better flexibility compared to PLA but requires higher extrusion temperatures and takes longer to degrade. PVA is hydrophilic and water-soluble, making it a good candidate for applications involving hydrophilic drug delivery, whereas the hydrophobicity of PLA limits its compatibility with such APIs. However, the mechanical strength and biocompatibility of PLA make it a better choice for applications where structural integrity and skin safety are essential [24].

PLA is generally well-suited for FDM due to its relatively low melting point, ease of processing, and mechanical strength. Its use in dermatological preparations is feasible when the specific application benefits from its biodegradability and biocompatibility. However, for applications that require the incorporation of hydrophilic drugs, PLA may not be the ideal choice unless modified through the addition of plasticizers or blended with other polymers. Alternative 3D printing methods, such as Semi-Solid Extrusion (SSE) or Stereolithography (SLA), could offer advantages for PLA-based formulations where improved drug loading or more precise structures are required [55].

#### 4.3.6. Polyvinyl Alcohol (PVA)

**Figure 6:**
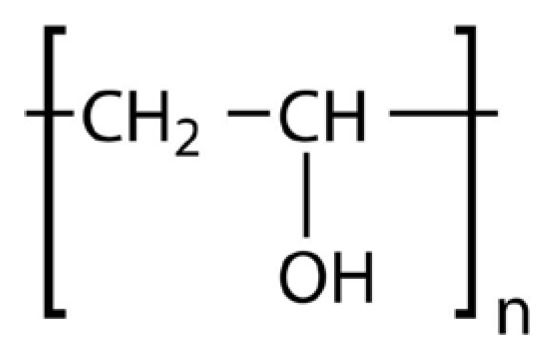
Chemical structure of PVA.

Polyvinyl Alcohol (PVA) is a hydrophilic polymer extensively employed for controlled and sustained drug release due to its water solubility. The hydroxyl groups in its molecular chain, depicted in Figure 6, facilitate controlled drug release but also make it susceptible to moisture absorption, impacting its stability during storage and printing.

General Formula: (C_2_H_4_O)_n_
Functional Groups:
  - Hydroxyl Groups (-OH):
    ▪ Provide strong hydrophilicity, making PVA water-soluble.
    ▪ Facilitate interaction with APIs and enhance compatibility.
  - Hydrocarbon Backbone (-CH_2_-):
    ▪ Contributes to limited hydrophobicity, improving interaction with hydrophobic APIs.

Physical Description:
  - Appearance: White to cream-colored granules or powder. [65]
  - Odor: Odorless. [65]
  - Melting Point: Decomposes > 200 °C [66].
  - Solubility: Fully soluble in water; slightly soluble in ethanol; insoluble in most organic solvents [66].

Impact on Stratum Corneum Permeation:
  - The hydrophilic hydroxyl groups in PVA hydrate the stratum corneum, enhancing drug permeability.
  - PVA forms a flexible matrix, making it suitable for drug-loaded films and patches in dermatological applications.

##### Properties of Polyvinyl Alcohol (PVA)

PVA is a biocompatible polymer, which makes it an ideal material for applications in direct contact with the skin, such as dermatological preparations, hydrogels, and even synthetic articular cartilage [67]. The non-toxic nature of PVA ensures its safety in transdermal drug delivery systems, minimizing the risk of irritation or adverse reactions. This characteristic is especially relevant for creating personalized drug delivery devices like 3D-printed patches, where direct and prolonged skin contact is required. Studies have shown that PVA has minimal irritation properties, making it suitable for use in medical applications [66,68].

The water solubility of PVA is one of its most notable characteristics, allowing it to dissolve easily in water without requiring any organic solvents. This property not only contributes to the polymer’s biodegradability, but also enhances its utility in FDM 3D printing for pharmaceutical applications. The ability of PVA to dissolve in water makes it an ideal choice for printing water-soluble support structures, which can be easily removed post-printing [66,68]. Additionally, its hydrophilic nature enhances drug release properties, making PVA-based 3D-printed formulations effective for sustained drug release. However, the hygroscopic nature of PVA requires careful handling to avoid moisture absorption, which can lead to printing inconsistencies [68].

PVA has a melting point of approximately 163 °C and a Vicat softening temperature of 60.2 °C, making it suitable for FDM 3D printing when processed within specific temperature ranges [66]. The viscosity of PVA influences its printability, with high viscosity ensuring consistent flow through the extruder nozzle, which is crucial for achieving smooth and accurate prints. The viscosity can be optimized by adding plasticizers, such as glycerol or sorbitol, to reduce processing temperatures and facilitate smooth extrusion [68]. This is particularly important for printing flexible transdermal patches or other dermatological devices that require both mechanical strength and flexibility.

The spreadability of PVA is closely related to its ability to form consistent layers during the FDM process. The good film-forming ability of PVA ensures that each printed layer adheres well to the previous one, resulting in structurally stable and robust printed objects. This property is beneficial for producing patches and other drug delivery devices that need to maintain integrity during application to the skin [68].

##### Advantages and Disadvantages of Using Polyvinyl Alcohol (PVA) in Fused Deposition Modeling (FDM)

PVA offers several advantages in FDM, including biocompatibility, ease of dissolution, and biodegradability. Its water solubility makes it easy to remove as a support material, and its non-toxic nature makes it suitable for use in drug delivery systems intended for prolonged contact with the skin [68]. Additionally, the film-forming properties of PVA and the ability to create hydrogels make it a versatile polymer for creating patches that deliver drugs directly to targeted areas [69].

However, there are also challenges associated with using PVA in FDM. Its hygroscopic nature makes it prone to absorbing moisture, which can lead to filament degradation and printing issues if not properly stored and handled. Additionally, the melting point of PVA requires careful temperature control during printing to avoid decomposition or jamming of the extruder nozzle [66].

The properties of PVA make it suitable for use in FDM 3D printing, particularly for creating personalized drug delivery devices. Its biocompatibility and water solubility are beneficial for transdermal and topical applications, where patient safety and ease of use are critical. However, to ensure optimal printability and structural integrity, PVA should be used in combination with plasticizers and handled carefully to avoid moisture absorption. The FDM method is well-suited for PVA, as it allows for precise control over the printing process, enabling the production of customized patches and drug delivery devices that fit the unique needs of individual patients [66,68].

#### 4.3.7. Chitosan

**Figure 7:**
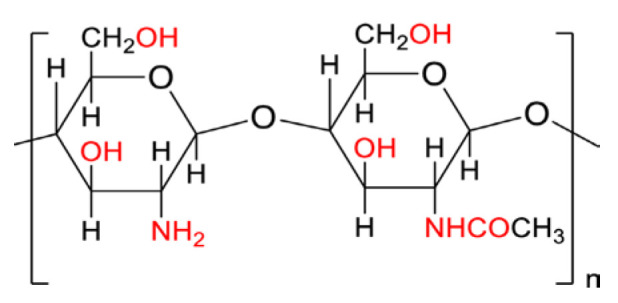
Chemical structure of chitosan.

Chitosan is recognized for its remarkable antimicrobial activity, biocompatibility, and bioadhesiveness, making it particularly effective in wound healing applications. The presence of reactive amine groups within its molecular structure, illustrated in Figure 7, supports these therapeutic properties.

General Formula: (C_6_H_11_NO_4_)_n_
Functional Groups:
  - Amino Groups (-NH_2_):
    ▪ Provide hydrophilicity by forming hydrogen bonds with water molecules.
    ▪ Enhance ionic interactions, improving API compatibility.
  - Hydroxyl Groups (-OH):
    ▪ Contribute to hydrophilicity and solubility in acidic environments.
    ▪ Allow for modifications to enhance solubility and API inclusion.
  - Glycosidic Linkages (-O-):
    ▪ Provide structural stability but contribute to limited hydrophobicity.
Physical Description:
  - Appearance: Off-white to beige powder [70].
  - Odor: Odorless [70].
  - Melting Point: Decomposes at approximately 102.5 °C [70].
  - Solubility: Soluble in dilute acids (e.g., acetic acid); insoluble in water and organic solvents at neutral pH [71,72,73].

Impact on Stratum Corneum Permeation:

Hydrophilic amino and hydroxyl groups enhance the hydration of the stratum corneum, improving drug permeation.

Forms a flexible and bio-adhesive matrix, making it suitable for drug delivery systems like films and gels [71].

##### Properties of Chitosan

Chitosan is a natural polysaccharide derived from chitin, commonly found in the exoskeletons of crustaceans such as crabs and shrimp [74]. Known for its remarkable biocompatibility, biodegradability, and antimicrobial properties, chitosan is widely used in a variety of biomedical applications, including drug delivery systems and wound dressings [1]. Its biocompatibility ensures minimal foreign body reaction and little to no fibrous encapsulation, which is advantageous for dermatological and wound healing applications [75,76]. Additionally, chitosan is biodegradable and can be broken down by enzymes such as lysozyme, a glycosidic hydrolase present in the human body, which hydrolyzes the β (1–4) linkages between N-acetylglucosamine and glucosamine [77,78]. This property makes chitosan particularly appealing for applications that require gradual degradation, such as topical patches or wound healing scaffolds. The controlled biodegradation allows for sustained release of therapeutic agents, reducing the need for patch removal and enhancing patient compliance. As an added benefit, Chitosan exhibits broad-spectrum antimicrobial activity and its effectiveness is influenced by factors such as molecular weight, degree of deacetylation, and pH of the environment [79].

Chitosan is soluble in acidic aqueous solutions due to the protonation of its amino groups, which gives it a positive charge and allows it to interact with negatively charged drugs and biological components. This solubility characteristic is advantageous for creating hydrogels and films for drug delivery. However, the requirement for acidic conditions to dissolve chitosan may present challenges in processing, especially for FDM, which generally operates at neutral pH [42]. The solubility profile of chitosan can impact its ability to be uniformly mixed with Active Pharmaceutical Ingredients (APIs), influencing the homogeneity and drug release characteristics of 3D-printed formulations [80].

Chitosan does not have a well-defined melting point, as it degrades before melting when exposed to high temperatures. This characteristic poses a significant challenge for its use in FDM, which relies on thermoplastic properties for filament extrusion. Instead of melting, chitosan undergoes thermal decomposition at temperatures above 200 °C, which makes it unsuitable for conventional FDM without modifications [42]. To overcome this limitation, chitosan can be blended with thermoplastic polymers or plasticizers to improve its thermal stability and make it more amenable to FDM processing [80].

The viscosity of chitosan solutions is influenced by factors such as molecular weight, degree of deacetylation, and concentration. High viscosity can be advantageous for creating stable 3D-printed structures, but it may also pose challenges during extrusion, as high-viscosity materials are more prone to clogging the printer nozzle [81]. The spreadability of chitosan in FDM is limited due to its high viscosity, which may impact the layer adhesion and overall print quality. Therefore, optimizing the viscosity through dilution or by adding plasticizers is necessary to improve its printability.

##### Advantages and Disadvantages of Using Chitosan in Fused Deposition Modeling (FDM)

Chitosan offers several advantages in 3D printing for dermatological applications. Its biocompatibility and biodegradability make it an attractive option for creating wound dressings, transdermal patches, and other skin-contact devices [76,80]. Additionally, its antimicrobial properties help prevent infections, which is particularly beneficial for wound healing applications. The positive charge of chitosan allows it to interact with negatively charged APIs, enhancing drug loading and providing sustained drug release [1].

However, there are significant limitations to using chitosan in FDM. Its lack of a defined melting point and thermal degradation at temperatures above 200 °C make it unsuitable for standard FDM processes [42]. The high viscosity of chitosan solutions can also create challenges during extrusion, resulting in poor layer adhesion and inconsistent print quality. To address these limitations, chitosan is often blended with other polymers, such as Polyvinyl Alcohol (PVA), or combined with plasticizers to improve its thermal and mechanical properties, making it more suitable for FDM [81].

While chitosan has several beneficial properties for biomedical applications, its use in FDM is limited due to its thermal degradation and high viscosity. To make chitosan suitable for FDM, it is often combined with other thermoplastic polymers or plasticizers to enhance its processability [42]. However, alternative 3D printing methods, such as Semi-Solid Extrusion (SSE) or Stereolithography (SLA), may be more suitable for chitosan-based formulations. SSE, for example, allows for the extrusion of chitosan-based pastes without requiring high temperatures, making it a better option for heat-sensitive materials [81]. SLA can also be used to create high-resolution chitosan-based structures by incorporating photocurable chitosan derivatives, which provides greater control over the geometry and mechanical properties of the printed objects.

#### 4.3.8. Hydroxypropyl Cellulose (HPC)

**Figure 8:**
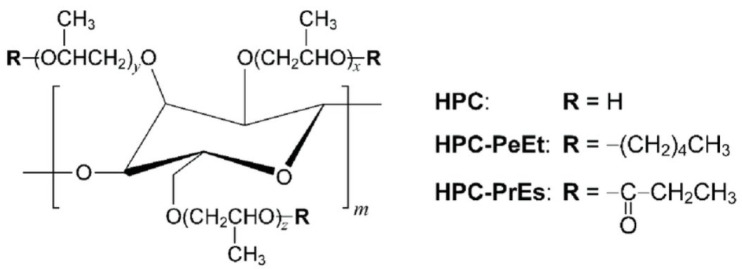
Chemical structure of HPC.

Hydroxypropyl Cellulose (HPC) is a cellulose derivative that excels in controlled and sustained drug release due to its hydrophilicity and film-forming properties. The cellulose-based structure shown in Figure 8 supports its effectiveness as a drug carrier, particularly for topical preparations requiring prolonged therapeutic action.

General Formula: (C_6_H_7_O_2_(OH)_x_(OCH_3_)_γ_)_n_, where _x_ and _γ_ vary based on hydroxypropyl substitution levels.
Functional Groups:
  - Hydroxyl Groups (-OH):
    ▪ Provide strong hydrophilicity, enhancing water solubility and drug compatibility.
    ▪ Facilitate hydrogen bonding with APIs and improves matrix formation.
  - Ether Groups (-O-):
    ▪ Contribute to moderate hydrophobicity, enhancing interaction with certain hydrophobic APIs.
    ▪ Offer structural flexibility and chemical stability.

Physical Description:
  - Appearance: White or off-white powder. [82]
  - Odor: Odorless. [82]
  - Melting Point: Decomposes without a defined melting point [83].
  - Solubility: Soluble in water and organic solvents like ethanol; solubility increases with higher temperatures and specific pH conditions.

Impact on Stratum Corneum Permeation:
  - Hydrophilic hydroxyl groups hydrate the stratum corneum, enhancing drug permeation.
  - Ether and hydroxyl functionalities contribute to bioadhesion and controlled release, making it suitable for dermatological films and transdermal patches.

##### Properties of Hydroxypropyl Cellulose (HPC)

Hydroxypropyl Cellulose (HPC) is a water-soluble cellulose derivative used extensively in pharmaceutical applications, particularly for drug delivery systems. HPC is synthesized by reacting alkali cellulose with propylene oxide, which substitutes hydroxyl groups on each anhydrous monomer unit of cellulose through ether linkage. It is available in different grades that vary in viscosity and molecular weight, ranging from 40,000 to 1,150,000 Daltons [84]. HPC is known for its excellent film-forming properties, making it suitable for creating solid dispersions and enhancing the bioavailability of poorly water-soluble drugs. The application of HPC in 3D printing, especially in combination with Hot Melt Extrusion (HME), allows for the fabrication of customized dosage forms tailored to individual patient needs [84,85].

HPC is biocompatible and widely used in drug delivery applications that require direct contact with biological tissues. Its non-toxic nature ensures that it can be used safely in dermatological applications, such as transdermal patches and other topical drug delivery devices. This biocompatibility makes HPC an ideal candidate for personalized treatments that require precise and controlled drug release for skin-related ailments [84].

HPC is soluble in both cold and hot water, as well as in many organic solvents. This property facilitates the formation of homogenous mixtures with drugs, which is critical for achieving consistent drug distribution in printed formulations. The solubility of HPC also enables the formation of gels and hydrogels, providing a controlled drug release mechanism [84]. However, its solubility may present challenges during the 3D printing process, particularly if the polymer absorbs moisture from the environment, potentially leading to compromised filament quality and print consistency.

The glass transition temperature (Tg) of HPC is approximately 120 °C. This high Tg helps maintain the structural stability of printed objects, but also necessitates high processing temperatures during extrusion and printing, which could limit its use with temperature-sensitive Active Pharmaceutical Ingredients (APIs) [84]. In order to achieve lower extrusion and printing temperatures, plasticizers are often added, reducing the Tg of HPC and improving its printability [84].

HPC exhibits high viscosity, which is beneficial during the extrusion process for producing consistent filaments. This viscosity also contributes to good spreadability and layer adhesion in printed structures, ensuring that the final product has a smooth surface and minimal defects. The mechanical properties of HPC, such as its high viscosity and gel-forming capability, are critical for applications in dermatology where controlled drug release is essential [84].

##### Advantages and Disadvantages of Using Hydroxypropyl Cellulose (HPC) in Fused Deposition Modeling (FDM)

One of the major advantages of HPC in Fused Deposition Modeling (FDM) is its ability to form homogeneous blends with APIs, enhancing drug solubility and bioavailability. Its biocompatibility ensures that it can be used safely for dermatological applications. The capability of HPC to form hydrogels makes it suitable for 3D-printed transdermal patches that require sustained drug release [84].

However, HPC has limitations in FDM. The high Tg requires elevated processing temperatures, which may not be suitable for all drugs, especially those sensitive to heat. Furthermore, the hygroscopic nature of HPC can lead to issues with filament stability and printability if not properly stored and processed [84].

HPC is suitable for use in FDM, particularly when combined with plasticizers to lower its Tg and improve its processing characteristics. The combination of hot melt extrusion and FDM has been shown to be effective for the production of HPC-based floating tablets of cinnarizine, where the floating force and drug release profile were controlled by printing parameters [84]. This demonstrates the versatility of HPC in creating tailored dosage forms for personalized medicine.

However, for formulations requiring lower processing temperatures or high-resolution printing, other 3D printing techniques like inkjet printing or stereolithography (SLA) might be more appropriate. Inkjet printing is a non-thermal process, making it ideal for heat-sensitive APIs, while SLA can achieve finer resolution, which is beneficial for creating intricate drug delivery devices [84].

#### 4.3.9. Polycaprolactone (PCL)

**Figure 9:**
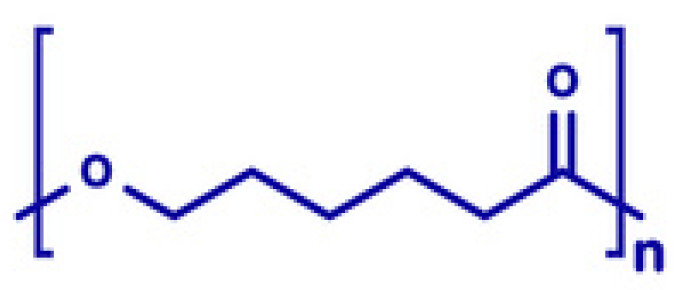
Chemical structure of PCL.

Polycaprolactone (PCL) is a biodegradable and flexible polymer suitable for long-term drug delivery and tissue engineering applications. Its molecular arrangement, depicted in Figure 9, allows for low-temperature extrusion, making it ideal for heat-sensitive APIs, despite limitations with hydrophilic drug loading.

General Formula: (C_6_H_10_O_2_)_x_
Functional Groups:
  - Ester Group (-COO-): Provides strong hydrophobicity, limiting water solubility and enhancing compatibility with hydrophobic APIs.
  - Hydrocarbon Backbone (-CH_2_-): Contributes to flexibility and chemical stability, supporting the inclusion of various APIs.

Physical Description:
  - Appearance: Clean white, semi-crystalline solid. [86]
  - Odor: Odorless.
  - Melting Point: ~60 °C.
  - Glass Transition Temperature: −60 °C.
  - Solubility: Insoluble in water; soluble in organic solvents like chloroform and benzene. [86]

Impact on Stratum Corneum Permeation:
  - Hydrophobic ester groups promote controlled drug release and enhance interaction with lipid-rich stratum corneum.
  - Provides flexibility and structural stability, making it ideal for applications like wound dressings and transdermal patches [87].

##### Properties of PCL

Polycaprolactone (PCL) is a biodegradable polyester that has gained significant attention in the field of additive manufacturing due to its favorable properties for tissue engineering and pharmaceutical applications [88,89]. Its low melting point (approximately 60 °C) makes it particularly suitable for Fused Deposition Modeling (FDM), allowing for the creation of intricate structures while maintaining low energy requirements [41].

PCL is highly biocompatible, which is crucial for applications involving direct contact with biological tissues, such as dermatological drug delivery systems. Its compatibility with living tissues is well-documented, and it has been approved by the FDA for various biomedical applications, making it a suitable candidate for dermatological preparations [41]. Additionally, the biocompatibility of PCL is particularly important in wound healing and tissue regeneration, where direct interaction with biological tissues is essential [90].

PCL is hydrophobic, which limits its ability to carry hydrophilic drugs. However, it has been effectively used for sustained drug release due to its slow degradation rate, which makes it ideal for long-term applications such as wound dressings or dermatological patches. The hydrophobicity of PCL can be managed by blending it with hydrophilic polymers or hydrogels to enhance its drug loading and release properties, thereby overcoming this limitation [91].

The low melting point of PCL, which is around 55–60 °C, makes it suitable for FDM, as it allows for extrusion at lower temperatures without the risk of degrading sensitive Active Pharmaceutical Ingredients (APIs) [41]. This property makes PCL an excellent choice for creating drug delivery systems that need to maintain the stability of the loaded drugs during processing. Its viscosity is also favorable for 3D printing, allowing for smooth extrusion through the nozzle, which contributes to the consistent quality of printed products.

PCL has good spreadability, which enhances the adhesion between layers during the printing process. This property is crucial for achieving structural stability and ensuring the mechanical robustness of the final printed objects. The ability of PCL to form strong interlayer bonds results in stable and durable 3D-printed structures, which is particularly beneficial in applications that require prolonged contact with the skin, such as wound dressings [41].

##### Advantages and Disadvantages of Using Polycaprolactone (PCL) in Fused Deposition Modeling (FDM)

PCL is ideal for FDM-based drug delivery due to its biocompatibility, low melting point, and mechanical flexibility. Its high elongation capacity makes it suitable for wound dressings and dermatological patches, conforming to irregular surfaces [41]. Additionally, its slow degradation rate supports sustained drug release, making it effective for long-term therapeutic applications.

However, the hydrophobic nature of PCL limits its ability to load and release hydrophilic drugs. It also lacks inherent bioactivity, requiring blending with bioactive materials or surface modifications to improve cellular interaction and tissue regeneration [41].

Compared to PLA and PEG, PCL offers greater flexibility and controlled degradation. While PLA is biodegradable and mechanically strong, it is brittle and requires plasticizers for flexibility. In contrast, the natural flexibility of PCL makes it more suitable for wound care products [41]. Additionally, the low melting point of PCL eliminates the need for plasticizers, simplifying FDM printing and reducing additives [91].

The low extrusion temperature of PCL allows for incorporating heat-sensitive APIs into FDM filaments. However, its hydrophobicity may require blending or composite formulations to enhance drug loading and release for dermatological applications [41].

While FDM is the most common printing method for PCL, other techniques like Melt Electrospinning Writing (MEW) and Selective Laser Sintering (SLS) offer additional benefits. MEW enables the creation of PCL scaffolds with high precision, which are useful for tissue engineering applications that require specific structural properties and high surface area [41].

#### 4.3.10. Thermoplastic Polyurethanes (TPUs)

**Figure 10:**
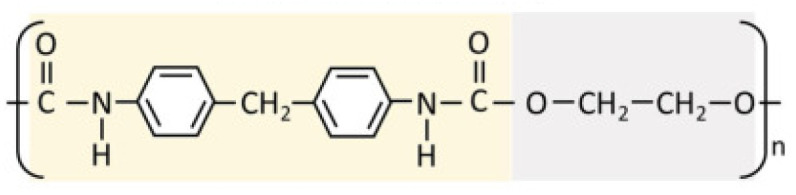
Chemical structure of TPU.

Thermoplastic Polyurethane (TPU) is known for its exceptional flexibility, durability, and biocompatibility, making it ideal for wearable dermatological applications such as flexible transdermal patches. Its structural properties, notably high tensile strength and elongation, are demonstrated in Figure 10, facilitating applications requiring adaptability to irregular skin surfaces.

Composition: TPU is a block copolymer with alternating hard segments (e.g., isocyanates) and soft segments (e.g., polyols like poly(ethylene oxide) or poly(tetrahydrofuran)) [92,93].
Functional Groups:
  - Urethane Groups (-NHCOO-): Provide flexibility, resilience, and chemical resistance, enhancing compatibility with APIs.
  - Ester/Ether Groups (in soft segments): Contribute to hydrophilicity (e.g., poly(ethylene oxide)) or hydrophobicity (e.g., poly(tetrahydrofuran)).
  - Hydrocarbon Chains (from polyols and isocyanates): Enhance hydrophobicity and mechanical strength.

Physical Description:
  - Appearance: Semi-transparent, rubber-like solid [93].
  - Odor: Odorless.
  - Melting Temperature: TPU does not have a sharp melting point but softens over a range of temperatures depending on its formulation [92].
  - Solubility: Insoluble in water; resistant to oils, greases, and most industrial solvents [93].

Impact on Stratum Corneum Permeation:
  - The combination of hydrophilic and hydrophobic segments of TPU allows for the controlled release of APIs and improved bioadhesion.
  - The flexibility of TPU enhances conformability to the skin, making it ideal for transdermal drug delivery systems [93].

##### Properties of Thermoplastic Polyurethane (TPU)

Thermoplastic Polyurethanes (TPUs) are versatile materials that are well-suited for 3D printing, particularly using Fused Deposition Modeling (FDM). They are known for their unique combination of rubber and plastic-like properties, offering flexibility, biocompatibility, and chemical resistance. These properties make TPU an attractive candidate for fabricating dermatological preparations, including drug delivery systems and wound dressings [94].

TPU is considered biocompatible, which makes it suitable for medical and dermatological applications where prolonged contact with the skin is required. The biocompatibility of TPU has been demonstrated through in vitro studies, which show minimal cytotoxic effects when tested with various cell lines [95]. This property ensures the safety of TPU-based 3D-printed medical devices, making it a suitable material for applications like wound dressings and drug delivery devices.

TPU is not water-soluble, which can be advantageous for creating durable structures that must withstand exposure to moisture. However, TPU is soluble in organic solvents, which allows for certain fabrication techniques beyond FDM, including solvent casting.

The melting point of TPU varies depending on its formulation, but the typical melting temperature for TPU used in 3D printing is around 220–240 °C [96]. This relatively high melting point allows TPU to maintain thermal stability during the FDM process, which is crucial for forming precise and consistent 3D structures. The melting point also influences the energy requirements of the extrusion process, and the ability of TPU to withstand higher temperatures ensures robust bonding between layers during printing.

TPU has a suitable viscosity for FDM, which ensures consistent extrusion and proper flow during the printing process. Its inherent flexibility also contributes to strong interlayer bonding, reducing the risk of delamination and enhancing the overall mechanical properties of printed parts [95]. The combination of good viscosity and spreadability also ensures that TPU can be extruded smoothly, producing accurate layer deposition and minimizing defects in printed structures.

The flexibility of TPU contributes to excellent interlayer bonding, which is critical in FDM printing. TPU filaments can produce printed parts with strong mechanical integrity, which is particularly beneficial for flexible drug delivery devices and wearable patches. The tensile properties of TPU, characterized by a tensile stress at break of 39 MPa and an elongation at break of up to 580%, showcase its mechanical durability and elasticity [96]. These properties enable TPU to accommodate movement and deformation, making it ideal for use in patches that adhere to the skin, where flexibility and comfort are essential.

##### Advantages and Disadvantages of Using Thermoplastic Polyurethane (TPU) in Fused Deposition Modeling (FDM)

The flexibility of TPU makes it ideal for personalized dermatological patches and wound dressings. It has high tensile strength and elongation at break, allowing it to support load-bearing applications while maintaining flexibility. Additionally, its resistance to industrial oils and chemicals enhances the durability of printed devices exposed to such substances [95].

However, the flexibility of TPU poses printing challenges, such as filament buckling during extrusion. Specialized print heads and modified settings are often needed for smooth processing. Additionally, TPU absorbs moisture, which can lead to printing defects like bubbles and weak spots in finished products.

TPU is well-suited for FDM due to its thermal stability and flexibility, but printer modifications are often required. Alternative methods like Direct Ink Writing (DIW) and Selective Laser Sintering (SLS) offer greater versatility. DIW enables the direct extrusion of viscous materials, allowing for incorporation of active pharmaceutical ingredients. SLS sinters powdered TPU using a laser, making it ideal for intricate geometries that FDM struggles to achieve.

#### 4.3.11. Eudragit

**Figure 11:**
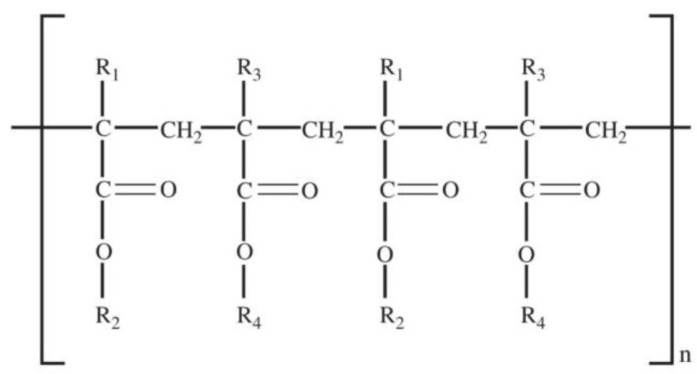
Chemical structure of Eudragit.

Eudragit is a polymer widely recognized for its excellent biocompatibility and versatility in drug release modulation. It varies by grade, typically copolymers of methacrylic acid and its esters or aminoalkyl methacrylate. Its general molecular structure is shown in Figure 11.

General composition:
Varies by grade, typically copolymers of methacrylic acid and its esters or aminoalkyl methacrylate [97,98].
Functional Groups:
  - Ester Groups (-COO-): Provide hydrophobicity, reducing solubility in water and enabling sustained release [98].
  - Carboxylic Acid Groups (-COOH): Enhance hydrophilicity and pH-dependent solubility, facilitating targeted drug release in gastrointestinal regions [97].
  - Amino Groups (-NH_2_ or -N(CH_3_)_2_): Contribute to cationic properties, improving drug compatibility and solubility in acidic pH [97].

Physical Description:
  - Appearance: Available as powders, granules, aqueous dispersions, or organic solutions [97].
  - Odor: Mild to odorless, depending on the grade [97].
  - Solubility: Soluble in organic solvents and specific pH conditions; insoluble in neutral or basic pH for some grades [98].

Impact on Stratum Corneum Permeation:
  - Cationic properties improve adhesion to negatively charged skin surfaces, aiding in transdermal drug delivery [97].

##### Properties of Eudragit

Eudragit is a versatile family of methacrylate-based copolymers widely used in pharmaceutical applications, especially for controlled drug release [97,99]. Its pH-sensitive and versatile nature makes it a popular choice for drug delivery systems. Eudragit polymers, including Eudragit E100, RL, and RS, have gained attention in the context of Fused Deposition Modeling (FDM) 3D printing due to their ability to form filaments that can be used to produce personalized drug formulations.

Eudragit grades are distinguished by their glass transition temperature (Tg), X-ray particle diffraction, DSC, FT-IR spectra, physiological buffer interactions, and pH-sensitive properties. A differential thermal study reveals a single Tg for each grade, impacting storage, film production, and melt processing in pharmaceutical applications. The amorphous nature of Eudragit facilitates prolonged drug release, and the addition of small molecules like drugs, solvents, or plasticizers can lower the Tg, enhancing its applicability. Triethyl citrate is commonly used as a plasticizer in Eudragit formulations [98].

X-ray diffractograms of grades such as Eudragit S 100, L 100, RS, and RL confirm their amorphous nature. TGA and DSC studies of Eudragit L30D, L, and S show thermal characteristics that align with FT-IR reflectance data. FT-IR spectra for these polymers exhibit characteristic ranges: C-H stretching (3100–2850 cm^−1^), C=O stretching (1800–1650 cm^−1^), and C-O stretching (1350–900 cm^−1^). The diffraction patterns show a halo, further supporting their amorphous structure. These properties underline Eudragit’s versatility in pharmaceutical formulations [98]. Below are some examples of Eudragit with their individual properties:

Eudragit E (EE):
  - Cationic copolymer soluble at gastric pH ≤ 5.
    ▪ Fast dissolution due to the hydration of protonated dimethylamino groups.
    ▪ Used for solid dispersions (SDs), sublingual and topical preparations, and modified-release tablets.
  - Eudragit RL (ERL):
    ▪ Permeable, cationic polymer with 10% quaternary ammonium groups.
    ▪ pH-independent swelling and high permeability.
    ▪ Composed of methyl methacrylate, ethyl acrylate, and methacrylic acid ester.
    ▪ Chemically stable, excellent extrudability, and insoluble in water.
    ▪ Used in micro/nanoparticles, coated tablets, and sustained-release systems.
  - Eudragit RS (ERS):
    ▪ Similar to ERL but with lower permeability (5% quaternary ammonium groups).
    ▪ Often blended with ERL to achieve specific permeability and absorption rates.
    ▪ Used in sustained-release systems like mucoadhesive films and coated tablets.
  - Eudragit S100 (ES100), L100 (EL100), and L100-55 (EL100-55):
    ▪ Anionic polymers with varying carboxylic group contents:
      ♦ ES100: Soluble above pH 7.0; 29.2% carboxylic groups.
      ♦ EL100: Soluble above pH 6.0; 48.3% carboxylic groups.
      ♦ EL100-55: Soluble above pH 5.5; copolymer of methacrylic acid/ethyl acrylate.
    ▪ Commonly used for enteric coatings.
  - Eudragit FS 30 D (EFS30D):
    ▪ Anionic polymer composed of methyl acrylate, methyl methacrylate, and methacrylic acid.
    ▪ Available as a 30% aqueous dispersion with low viscosity.
    ▪ Soluble above pH 7.0; used for colonic drug delivery systems.

Eudragit polymers are generally biocompatible, which makes them suitable for dermatological applications where direct contact with the skin is required. Eudragit E100, for instance, is cationic and soluble in acidic environments, making it useful for formulations intended for specific pH conditions [100]. This pH-dependent solubility allows for controlled drug release, which is particularly advantageous for transdermal or topical applications where targeted drug delivery is desired. However, the solubility properties of Eudragit RL and RS are pH-independent, making them suitable for sustained drug release across different conditions [101].

Eudragit polymers have a wide range of melting points and thermal properties, which are crucial in FDM 3D printing. The melting point of Eudragit E100 is around 110–130 °C, which allows it to be used in Hot Melt Extrusion (HME) to produce printable filaments [100]. The thermal stability of these polymers is also important to ensure that they do not degrade during the high-temperature extrusion process, maintaining the integrity of both the polymer and the Active Pharmaceutical Ingredient (API).

The viscosity of Eudragit-based filaments plays a vital role in their printability. The addition of plasticizers, such as Triethyl Citrate (TEC), helps to reduce the viscosity, making the material easier to extrude during the printing process [101]. Eudragit RL and RS have been used with plasticizers to improve their flexibility and reduce brittleness, which is often an issue with these polymers. The spreadability of Eudragit-based filaments also ensures that the printed layers adhere well, contributing to the robustness and consistency of the final printed product.

Eudragit polymers exhibit good mechanical properties, which are important for maintaining the structural integrity of 3D-printed dosage forms. The brittleness of Eudragit E100 can be mitigated by using additives like magnesium silicate and plasticizers such as triethyl citrate, which improve the flexibility and mechanical strength of the printed filaments [101].

##### Advantages and Disadvantages of Using Eudragit in Fused Deposition Modeling (FDM)

One of the main advantages of Eudragit polymers in FDM is their ability to provide controlled or sustained drug release. The pH-dependent solubility of Eudragit E100 makes it ideal for site-specific drug delivery, while Eudragit RL and RS are used for sustained release due to their pH-independent properties [101]. Eudragit polymers are also biocompatible and safe for dermatological use, which is critical for topical drug delivery systems.

However, the major drawback of Eudragit polymers in FDM is their brittleness, especially in the case of Eudragit E100. This brittleness makes it difficult to produce flexible and functional filaments, which is essential for continuous printing in FDM. The addition of plasticizers and other excipients can mitigate this issue but may alter the mechanical properties and the drug release profile of the final product. Moreover, Eudragit requires high processing temperatures, which may not be suitable for temperature-sensitive APIs.

Eudragit is suitable for FDM 3D printing, especially when combined with plasticizers to reduce brittleness and improve flexibility. The use of Hot Melt Extrusion (HME) for filament production helps to ensure that the filaments have consistent properties, which is essential for successful FDM printing. However, due to the brittleness of some Eudragit variants, other 3D printing methods such as direct ink writing or semi-solid extrusion may also be considered. These methods allow for the extrusion of semi-solid materials at lower temperatures, reducing the risk of API degradation and improving the overall flexibility of the printed product [101].

#### 4.3.12. Ethylene Vinyl Acetate (EVA)

**Figure 12:**
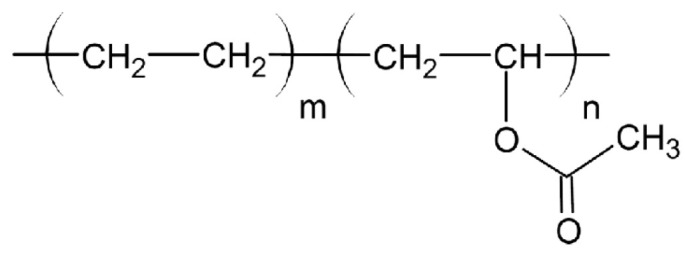
Chemical structure of EVA.

Ethylene Vinyl Acetate (EVA) combines flexibility with strong mechanical properties, making it suitable for flexible, conformable dermatological patches. The molecular structure provided in Figure 12 demonstrates its compatibility with various drug formulations and sustained-release capability, despite limitations with hydrophilic APIs.

General Formula: (C_4_H_6_O_2_)
Functional Groups:
  - Ester Groups (-COO-):
    ▪ Provide moderate hydrophilicity and compatibility with various APIs.
    ▪ Contribute to controlled drug release by interacting with hydrophilic APIs.
    ▪ Hydrocarbon Chains (Ethylene Units):
    ▪ Contribute to hydrophobicity and mechanical strength.

Physical Description:
  - Appearance: Translucent to opaque thermoplastic resin [102].
  - Odor: Odorless [103].
  - Melting Point: Approximately 90 °C, varying based on vinyl acetate content [102].
  - Solubility: Insoluble in water; soluble in organic solvents such as chloroform and toluene [103].

Impact on Stratum Corneum Permeation:
  - Compatible with APIs due to ester groups, allowing for gradual and sustained drug release [102].

##### Properties of Ethylene Vinyl Acetate (EVA)

Ethylene Vinyl Acetate (EVA) is a thermoplastic copolymer consisting of ethylene and vinyl acetate, known for its versatility and biocompatibility [104]. EVA is widely used in various biomedical applications, including drug delivery systems, due to its mechanical flexibility and ease of processing. A study by Brandl et al. [105] presents a novel filament fabrication method enabling 3D printing of personalized implants using elastic ethylene vinyl acetate copolymer, highlighting its potential in biomedical applications. In the field of 3D printing, EVA is often utilized in Fused Deposition Modeling (FDM) for the creation of drug delivery devices, particularly transdermal patches and topical medical applications [106].

EVA has excellent biocompatibility, which makes it highly suitable for biomedical applications, including drug delivery. The material is non-toxic and approved by the FDA for various medical applications, such as implants and drug delivery devices. This biocompatibility is critical for dermatological preparations, where prolonged contact with the skin is required, ensuring that EVA-based devices do not cause adverse reactions [107]. EVA has been used in medical-grade applications, including 3D-printed antiviral delivery devices, highlighting its safety and efficacy in biomedical settings [108].

EVA is a hydrophobic polymer with limited solubility in aqueous environments. Its hydrophobic nature can be advantageous for applications requiring controlled or sustained drug release, as it can effectively regulate the release profile of hydrophilic drugs [106]. The melting point of EVA can vary depending on the Vinyl Acetate (VA) content, typically ranging between 60 °C and 90 °C. A higher VA content results in a lower melting point, reduced stiffness, and enhanced flexibility, making EVA suitable for FDM applications that require low extrusion temperatures [106]. EVA-based intravaginal rings have also demonstrated this temperature-dependent flexibility, which is crucial for maintaining comfort and effective drug release [107].

EVA exhibits good viscosity characteristics, making it easy to process using FDM. Its viscosity allows for smooth extrusion and good layer adhesion during the printing process. Spreadability is also an important characteristic of EVA, as it ensures even distribution of the polymer during printing, leading to uniform printed structures. This is particularly useful for dermatological preparations where a consistent layer thickness is crucial for effective drug delivery [106]. The spreadability and viscosity of EVA also contribute to its use in personalized transdermal patches, which require consistent deposition of drug-loaded layers to ensure optimal therapeutic efficacy [106].

##### Advantages and Disadvantages of Using Ethylene Vinyl Acetate (EVA) in Fused Deposition Modeling (FDM)

EVA is ideal for FDM-based drug delivery due to its flexibility, mechanical strength, and sustained drug release. It is well-suited for transdermal patches and topical drug delivery devices, conforming to skin contours and enabling prolonged therapeutic effects with fewer applications [107]. In contrast to other polymers, EVA does not require plasticizers, simplifying formulation and enhancing stability [106]. Additionally, its good processability as filaments, pellets, and powders allows for high drug-loading in 3D-printed tablets, highlighting its versatility in pharmaceutical applications [109].

However, the hydrophobic nature of EVA limits drug loading, particularly for hydrophilic APIs. The two-step FDM process (filament preparation and printing) may expose thermosensitive drugs to high temperatures, risking degradation. Direct Powder Extrusion (DPE) has been proposed as an alternative, allowing for direct powder printing, reducing thermal stress on active compounds [106]. Slonov et al. [108] demonstrated that EVA-based transdermal patches produced via DPE effectively delivered personalized therapies, offering a promising solution to FDM-related limitations.

The biocompatibility, flexibility, and low melting point of EVA make it highly suitable for dermatological applications. However, its hydrophobicity and drug-loading challenges must be addressed. Combining EVA with excipients or alternative printing methods like DPE can enhance drug stability and formulation efficiency [106,108].

While EVA performs well in FDM, DPE offers advantages by eliminating filament preparation and reducing thermal degradation of thermosensitive drugs. In contrast to FDM, the DPE formulation is controlled by screw rotation, offering greater flexibility in material selection and drug delivery design [106].

## 5. Comparison of Polymers

Every polymer discussed above has unique properties that make it suitable for specific dermatological applications using FDM. PCL and TPU offer flexibility, making them ideal for wound dressings and patches, while PVA and HPC are advantageous for their solubility and biocompatibility, which are crucial for drug delivery. EVA provides flexibility and ease of processing but is hydrophobic, which may limit drug loading. Eudragit stands out for its controlled drug release properties but requires modification to reduce brittleness for FDM use. PEG, while not ideal as a primary polymer, is valuable as a plasticizer to enhance the flexibility of more rigid materials. The table below (Table 3) illustrates and summarizes the properties of various polymers for easier comparison. The choice of filament for FDM in dermatological applications should be based on the specific requirements of the drug delivery system, including drug compatibility, release profile, and mechanical properties [110].

When selecting a polymer for Fused Deposition Modeling (FDM) 3D printing in pharmaceutical topical drug delivery applications, there are several critical factors to consider to ensure optimal filament performance and drug delivery efficiency:

- Compatibility with Active Pharmaceutical Ingredient (API)
- Drug-Loading Capacity and Distribution
- Thermal Stability of the Polymer
- Mechanical Properties of Filaments
- Biodegradability and Biocompatibility
- Printability and Processing Parameters
- Drug Release Profile and Target Application

(a) Compatibility with Active Pharmaceutical Ingredient (API)

The compatibility of the polymer with the Active Pharmaceutical Ingredient (API) is one of the critical factors in polymer selection. The polymer must not only be capable of incorporating the API, but must also protect it from degradation during the printing process. For instance, during FDM, polymers are melted and extruded at temperatures between 60 and 250 °C, which can potentially degrade heat-sensitive APIs. Polymers like Polycaprolactone (PCL) have a low melting point (~60 °C), making them suitable for APIs that are unstable at higher temperatures [69]. The chemical structure of the API also plays a role—whether it is hydrophobic or hydrophilic will affect its compatibility with the polymer matrix. For example, the hydrophobic nature of PLA might not be suitable for hydrophilic drugs without modifying the polymer to enhance hydrophilic interactions [24].

(b) Drug-Loading Capacity and Distribution

The drug-loading capacity of the polymer is crucial, especially for topical applications where therapeutic efficacy depends on sufficient drug release to the skin. Polymers must allow for homogeneous drug distribution throughout the filament to ensure consistent drug release profiles. Chitosan, for example, has a high drug binding capacity due to its positive charge, which can form ionic interactions with negatively charged drugs, leading to better incorporation and controlled release [1]. On the other hand, highly crystalline polymers may result in uneven drug distribution during hot melt extrusion, which could compromise the drug release profile [37].

(c) Thermal Stability of the Polymer

Thermal stability is another factor that directly impacts the selection of polymers for FDM printing. The polymer must have a melting or glass transition temperature that supports FDM without degrading the API. For example, Polyvinyl Alcohol (PVA) has a suitable melting temperature range (180–220 °C) that can process APIs without significant degradation [69]. However, polymers like Polyvinylpyrrolidone (PVP) may require plasticizers to reduce their processing temperature, as they are prone to degradation at high temperatures [27].

(d) Mechanical Properties of Filaments

The mechanical properties of the filaments, including strength, flexibility, and toughness, are essential for ensuring the printability and final structure of drug delivery devices. For instance, Thermoplastic Polyurethane (TPU) is known for its flexibility, making it suitable for topical patches that need to conform to the skin’s surface without causing discomfort [26]. On the other hand, PLA provides high rigidity and structural integrity, making it a good choice for devices requiring stable drug release, but less suitable for applications requiring flexible, skin-conforming patches. The filament’s mechanical strength must also be sufficient to withstand the extrusion process without breaking or deforming [30].

(e) Biodegradability and Biocompatibility

Biodegradability and biocompatibility are vital factors, especially for polymers used in pharmaceutical applications. The selected polymer should ideally degrade into non-toxic products that are safely metabolized by the body. For topical drug delivery, polymers such as PLA and chitosan are popular due to their biocompatibility and biodegradability, which ensure that they do not cause irritation or adverse reactions when applied to the skin [36]. For tissue engineering, an ideal scaffold should be biocompatible, biodegradable, and possess mechanical properties similar to the tissue it aims to replace [111]. Chitosan, in particular, has antimicrobial properties, which adds value for wound healing applications [112], whereas the biodegradability of PLA makes it suitable for transient applications where the device should safely degrade over time.

(f) Printability and Processing Parameters

The ease of processing and printability of the polymer are practical considerations that influence the efficiency of device manufacturing. Polymers must have rheological properties compatible with the FDM process. They need to flow well when melted, adhere to the build platform, and solidify appropriately to retain the intended design. PVA, for example, is known for its good printability and water solubility, making it suitable as a support material in complex drug delivery structures [113]. The print and bed temperature must also be considered to ensure the printed layers adhere well, and the object maintains its shape throughout printing [110,114].

(g) Drug Release Profile and Target Application

The polymer selection should consider the desired drug release kinetics and the target application. For example, Eudragit RL is used for its ability to provide sustained drug release, which is ideal for applications where prolonged drug exposure is needed [115]. In contrast, the slow degradation rate of PCL makes it appropriate for long-term drug delivery applications, while fast-releasing drugs would require more water-soluble or porous materials like PVA or chitosan to achieve quick drug diffusion [39].

## 6. Applications of Three-Dimensional (3D) Printing in Dermatological Preparations

Three-dimensional printing offers a novel approach to create customized drug delivery systems for topical applications, providing precise control over drug dosing, spatial targeting, and release profiles [116]. These innovative methods help to address the limitations of traditional topical therapies, such as variability in drug absorption and poor patient compliance [30,116]. This article will explore the applications of 3D printing in dermatological preparations, highlighting specific uses such as patches, hydrogels, face masks, and microneedles, and discuss their benefits over conventional methods.

### 6.1. Three-Dimensional (3D)-Printed Patches and Transdermal Systems

Three-dimensional-printed patches are among the most promising applications in dermatology, providing targeted drug delivery with enhanced accuracy. In contrast to conventional patches, which are often generic and standardized, 3D-printed patches can be customized to conform to specific anatomical features, ensuring a snug fit and optimal contact with the skin [69]. For instance, 3D-printed nose patches have been developed to deliver drugs directly to inflamed or infected areas, such as those affected by acne or rosacea, allowing for localized treatment with minimal systemic side effects. The use of biocompatible materials such as Polylactic Acid (PLA) and Polyvinyl alcohol (PVA) ensures that these patches are safe for extended use without causing irritation [24].

Moreover, advanced multi-material 3D printing has enabled the creation of patches capable of delivering multiple drugs simultaneously. This capability allows for combining immediate and sustained release profiles within a single device, offering a more comprehensive treatment option for complex skin conditions, such as eczema or psoriasis [30]. Such multi-drug systems are particularly beneficial for conditions that require both rapid symptom relief and long-term maintenance therapy.

### 6.2. Three-Dimensional (3D)-Printed Hydrogels for Wound Healing

Hydrogels have long been used in wound care due to their ability to maintain a moist environment, which is crucial for effective healing. Three-dimensional printing allows for the customization of hydrogel-based wound dressings, which can be precisely tailored in terms of shape, size, and drug content [1]. Hydrogels created from biocompatible materials like PVA and chitosan are ideal for wound healing, as they not only provide a moist environment, but also deliver therapeutic agents such as antimicrobials, growth factors, or anti-inflammatory drugs [69]. For example, 3D-printed chitosan-based hydrogels have been shown to promote wound healing while providing antimicrobial protection, making them particularly useful in managing chronic wounds such as diabetic ulcers or burns [80]. The ability to incorporate multiple therapeutic agents within a single hydrogel matrix enhances the effectiveness of the treatment, allowing for both infection control and the promotion of tissue regeneration. The flexibility of 3D printing also allows for the production of hydrogels with varying porosities, enabling controlled release of the embedded drugs over time [117].

Three-dimensional printing is also being used in the development of 3D-printed hydrogel dressings for treating diabetic ulcers. These dressings, fabricated using hydrogel-based biomaterials, provide an optimal environment for wound healing by maintaining moisture, promoting cellular regeneration, and enabling controlled drug release [50]. A new class of high-performance, seaweed-based hydrogels for 3D printing developed by a team of researchers from North Carolina State University. Known as the self-reinforced Homocomposite Hydrogel (HHG), this material is created by integrating different-sized networks of identical alginates together resulting in a hydrogel reinforced with itself with enchanced mechanical properties [118]. Zhu et al. [44] reported a clinical trial where hydrogel dressings loaded with antimicrobial agents significantly improved wound closure rates and reduced infection risks in diabetic patients. The porous and flexible structure of these dressings ensures adherence to irregular wound surfaces while allowing for sustained release of therapeutic agents, thereby accelerating the healing process. Furthermore, the ability to customize drug concentration and release profiles makes 3D-printed hydrogel dressings an innovative solution for chronic wound management.

### 6.3. Face Masks and Personalized Skincare Products

Another exciting application of 3D printing in dermatology is the production of customized face masks and skincare products. Three-dimensional-printed face masks can be designed to fit the unique contours of an individual’s face, providing better adherence and improved efficacy of active ingredients [69]. These masks can be infused with a variety of active compounds, such as antioxidants, peptides, or anti-acne agents, which are precisely delivered to targeted areas of the skin [1]. The ability to tailor these face masks to individual anatomical features ensures that the formulation remains in contact with the desired area, enhancing the penetration of active ingredients and, subsequently, the therapeutic outcome.

The use of 3D printing in creating personalized face masks not only enhances the effectiveness of the treatment, but also significantly improves the user experience. Patients are more likely to comply with treatments that are comfortable, well-fitting, and designed specifically for their needs, making 3D-printed face masks an appealing option for targeted skincare solutions [69]. A beta version of Neutrogena’s custom 3D-printed sheet mask allows participants of this beta test use the Neutrogena Skin360 app to 3D scan their faces, creating precise facial maps that identify individual skin characteristics. The app then recommends customized skincare ingredients targeted precisely to each user’s unique skin concerns, like dryness or redness [119]. The final personalized mask is produced using a specialized 3D printing technique, placing active ingredients exactly where needed for optimal effectiveness [120].

Furthermore, the flexibility of 3D printing allows for the incorporation of biocompatible and biodegradable materials, ensuring safety for the skin and minimal environmental impact [1]. Such innovations are particularly relevant in personalized dermatological treatments, where the precision of drug delivery, combined with improved patient adherence, provides a significant advantage over traditional skincare solutions.

### 6.4. Three-Dimensional (3D)-Printed Microneedles for Transdermal Drug Delivery

Microneedles are another innovative application of 3D printing in dermatology. These tiny needles are designed to penetrate the stratum corneum, the outermost layer of the skin, to enhance the delivery of drugs into deeper layers without causing significant pain or discomfort [121]. Researchers in the United States have introduced a 3D micro-printed microneedle array that can painlessly deliver medications fabricated using biodegradable biomaterials designed to naturally break down within the patient’s body after drug administration [122]. The device holds significant potential for treating various medical conditions, including skin cancers. Three-dimensional printing allows for precise control over the size, shape, and arrangement of microneedles, which is crucial for optimizing drug delivery and minimizing skin damage.

Microneedles printed using Stereolithography (SLA) or Fused Deposition Modeling (FDM) have demonstrated improved drug penetration and patient outcomes compared to traditional methods [25]. The ability to customize the microneedle array to target specific skin conditions, such as localized inflammation or hyperpigmentation, makes this technology highly versatile. Furthermore, microneedles can be loaded with a wide range of therapeutic agents, including peptides, vaccines, and anti-inflammatory drugs, providing a minimally invasive yet effective solution for various dermatological issues.

Microneedle-based transdermal drug delivery systems have demonstrated promising results in clinical studies. Elkasabgy et al. [31] highlighted trials involving 3D-printed polymeric microneedle arrays, which effectively enhanced drug penetration through the stratum corneum, enabling systemic drug absorption with minimal invasiveness. These microneedles, designed with biodegradable and biocompatible polymers, dissolve upon application, eliminating the need for needle removal and reducing patient discomfort. Clinical findings suggest that 3D-printed microneedles can be particularly advantageous for pain-free vaccination, hormone therapy, and localized drug administration. Moreover, the precision of 3D printing allows for microneedles with varying lengths and drug-loading capacities, making them adaptable for different therapeutic needs. As research progresses, integrating personalized medicine and digital design in microneedle technology could revolutionize transdermal drug delivery in both dermatology and broader medical applications.

### 6.5. Future Prospects and Innovations

Three-dimensional printing technology continues to rapidly evolve presenting significant advancements for future medical applications. Recent developments have demonstrated the impact of 3D printing in personalized medicine and drug delivery systems. Personalized drug formulations created through 3D printing allow for precise dosing tailored to individual patient needs, significantly improving therapeutic outcomes and patient adherence [123]. The customization extends to complex drug delivery systems, such as polypills, which integrate multiple active pharmaceutical ingredients into a single dosage form, effectively simplifying treatment regimens [124,125,126].

Current innovations also include bioprinting, which involves printing living tissues and organoids by layering bio-inks containing cells. This technology is crucial for regenerative medicine, enabling the production of complex tissues and potentially functional organs, addressing shortages in donor organs and minimizing rejection risks through personalized cell sources [127]. Bioprinted tissues are also valuable for preclinical drug testing, providing accurate human tissue models that enhance the efficacy and safety evaluation processes of new drugs [123].

Further advancements in surgical preparations have been achieved through 3D-printed anatomical models. Surgeons utilize these models for preoperative planning, significantly reducing operative time and improving surgical outcomes by offering a tangible representation of patient-specific anatomy [126]. Additionally, the production of customized surgical instruments and prosthetics highlights the versatility of 3D printing, significantly lowering costs and shortening production times compared to traditional methods [127].

Looking towards the future, the integration of Artificial Intelligence (AI), 4D printing, and the Internet of Things (IoT) with 3D printing is expected to further revolutionize healthcare delivery. AI can streamline drug development and quality control processes, enhancing the predictability and efficiency of personalized medicine. Moreover, 4D printing, which involves creating structures that respond dynamically to environmental stimuli, is poised to produce smarter biomedical devices and implants that can adapt within the body over time [123,127]. The IoT will facilitate real-time monitoring and adaptive drug delivery through interconnected biomedical devices, marking a new era in patient-centered care and continuous therapeutic optimization [126]. Despite these promising developments, regulatory frameworks and quality control standards remain crucial factors that must evolve alongside technological advancements to ensure safety and efficacy in clinical settings [127]. Further research is needed to explore the full potential of 3D printing for topical drug delivery, including the development of new polymers, the optimization of printing parameters, and the integration of smart technologies that can provide real-time feedback on drug release and efficacy [113].

## 7. Conclusions

Three-dimensional printing technologies have emerged as powerful tools in advancing dermatological drug delivery systems. Fused Deposition Modeling (FDM) leverages diverse polymers, each offering unique properties for dermatological drug delivery. PLA provides biodegradability and structural integrity but requires modification for flexibility. PVA excels in controlled drug release due to its hydrophilicity but is sensitive to moisture. PCL is ideal for long-term applications with its flexibility and slow degradation but faces limitations with hydrophilic drugs. Chitosan offers antimicrobial benefits but struggles with thermal stability in FDM. PEG is used as a plasticizer to improve flexibility and lower extrusion temperatures. TPU is highly flexible, durable, and biocompatible rendering it suitable for wearable devices and long-term drug delivery systems. Other polymers, like HPC and Eudragit, support sustained and pH-sensitive release systems. By leveraging the strengths of different polymers and addressing their limitations through blending and the use of additives, FDM can facilitate the development of personalized and effective drug delivery systems that significantly improve patient outcomes in dermatology.

By leveraging the strengths of different polymers and addressing their limitations through blending and the use of additives, FDM can facilitate the development of personalized and effective drug delivery systems that significantly improve patient outcomes in dermatology. Recent advancements in polymer engineering have enabled sustained drug release through the strategic use of polymer blends, enhancing therapeutic efficacy and patient adherence [34,128]. Furthermore, biodegradable scaffolds show significant potential for skin regeneration, particularly in wound healing and tissue engineering applications, where 3D printing can create customized, bioactive structures to accelerate recovery [33]. However, regulatory challenges remain a critical hurdle in the widespread clinical adoption of 3D-printed dermatological products, necessitating further research and policy development to ensure safety, efficacy, and manufacturability in pharmaceutical applications [34,129]. Continued research and innovation are required to optimize these polymers for wider clinical use, overcoming existing limitations and expanding the applicability of 3D printing in pharmaceutical and biomedical fields.

## Figures and Tables

**Table 1:** Milestones of 3D printing.

Year	Milestone/Event
1981	First documented photopolymer rapid prototyping process by Hideo Kodama in Nagoya, Japan
1984	Unsuccessful French patent attempt on stereolithography by Alain Le Mehute, Olivier De Witte, and Jean Claude Andre
1984	Chuck Hull invents Stereolithography (SLA), pioneering commercial 3D printing
1988	Scott Crump invents Fused Deposition Modeling (FDM), enabling consumer-oriented 3D printing
1991	Commercialization of FDM technology by Stratasys
1992	Solidscape introduces the dot-on-demand printing method
1993	MIT partnership leads to significant advancements in inkjet-based 3D printing
1995	Fraunhofer Institute introduces Selective Laser Melting (SLM) for precision printing
1999	Wake Forest Institute applies 3D printing to produce scaffolds for biomedical applications
1999	First biomedical application: synthetic scaffold printed for organ support at Wake Forest
2001	Objet Geometries introduces first inkjet-based 3D printer
2002	Wake Forest researchers print first functional miniature kidney
2005	Launch of RepRap Project, making open-source FDM accessible
2008	First fully functional prosthetic leg printed via 3D printing
2009	Organovo successfully prints first functional blood vessel
2011	First 3D-printed car, Urbee, is successfully produced
2012	First 3D-printed gun sparks global security concerns
2014	China’s first fully 3D-printed house constructed, marking architectural breakthrough
2014	FDA approval of Spritam^®^, the first 3D-printed pharmaceutical product
2015	NASA explores 3D printing for medical applications in space
2016	Enhanced software enables mass-production and establishment of 3D printing farms
2016	Expansion of bioprinting technologies significantly improves drug delivery systems
2017	Novel approaches developed to optimize drug delivery and dosage accuracy
2019	Expansion into personalized medicine and complex pharmaceutical formulations
2022	Integration of AI and IoT for intelligent 3D-printed medical devices
2023	Advances in Direct Powder Extrusion (DPE) for improved pharmaceutical applications
2024	Further advancements in bioprinting towards printing fully functional organs and tissues

**Table 2:** Comparison of various 3D printing technologies.

No.	Technology	Resolution	Material Compatibility	Complexity of Post-Processing	Speed	Suitability for Thermolabile APIs
1	FDM	Moderate	Thermoplastics	Low	Fast	Moderate
2	SLA	High	UV-curable resins	High (UV-curing)	Moderate	Low (UV exposure)
3	SLS	High	Powder-based materials	Moderate (Powder removal)	Moderate	Low (High thermal stress)
4	DIW	Moderate	Viscous/Bio-inks	Moderate	Slow	High

**Table 3:** Comparison of various filaments, polymers, properties, and factors affecting the selection of polymers.

No.	Filament/Polymers	Moisture Resistance	Strength	Flexibility	Durability	Print Temperature (°C)	Bed Temperature (°C)
1	Polyethylene Glycol (PEG)	None	Moderate	High	Low	N/A	N/A
2	Kolliphor P188 (Poloxamer 188)	None	Moderate	High	Moderate	40–50	20–40
3	Polyvinylpyrrolidone (Kollidon 12PF)	Moderate	Moderate	Moderate	Moderate	150–180	40–60
4	Vinylpyrrolidone vinyl acetate copolymer (Kollidon VA64)	Moderate	High	Moderate	Moderate	180–210	60
5	Polylactic Acid (PLA)	Mild	High	Low	Moderate	190–220	60
6	Polyvinyl Alcohol (PVA)	None	Moderate	Moderate	Low	180–210	60
7	Chitosan	None	Moderate	Low	Low	N/A	N/A
8	Hydroxypropyl Cellulose (HPC)	Moderate	High	Moderate	Moderate	180–220	60
9	Polycaprolactone (PCL)	Strong	Moderate	High	High	60–120	25–40
10	Thermoplastic Polyurethanes (TPUs)	Moderate	High	Very high	High	210–230	40–60
11	Eudragit	Moderate to Strong	Moderate	Moderate	Moderate	150–180	40–60

## Data Availability

No new data were created or analyzed in this study. Data sharing is not applicable to this article.

## Abbreviations

3D	Three-Dimensional
API	Active Pharmaceutical Ingredient
FDM	Fused Deposition Modeling
PLA	Polylactic Acid
PVA	Polyvinyl Alcohol
PCL	Polycaprolactone
HPC	Hydroxypropyl Cellulose
PEG	Polyethylene Glycol
TPU	Thermoplastic Polyurethane
EVA	Ethylene Vinyl Acetate
SLA	Stereolithography
SLS	Selective Laser Sintering
SSE	Semi-Solid Extrusion
PEGDA	Polyethylene Glycol Diacrylate
Tg	Glass Transition Temperature
FDA	Food and Drug Administration
